# Astroglial toxicity promotes synaptic degeneration in the thalamocortical circuit in frontotemporal dementia with *GRN* mutations

**DOI:** 10.1172/JCI164919

**Published:** 2023-03-15

**Authors:** Elise Marsan, Dmitry Velmeshev, Arren Ramsey, Ravi K. Patel, Jiasheng Zhang, Mark Koontz, Madeline G. Andrews, Martina de Majo, Cristina Mora, Jessica Blumenfeld, Alissa N. Li, Salvatore Spina, Lea T. Grinberg, William W. Seeley, Bruce L. Miller, Erik M. Ullian, Matthew F. Krummel, Arnold R. Kriegstein, Eric J. Huang

**Affiliations:** 1 Department of Pathology,; 2 ImmunoX Initiative, and; 3 Eli and Edythe Broad Center of Regeneration Medicine and Stem Cell Research, UCSF, San Francisco, California, USA.; 4 Department of Neurobiology, Duke University School of Medicine, Durham, North Carolina, USA.; 5 Department of Ophthalmology, UCSF, San Francisco, California, USA.; 6 School of Biological and Health Systems Engineering, Arizona State University, Tempe, Arizona, USA.; 7 Neuroscience Graduate Program, UCSF, San Francisco, California, USA.; 8 Memory and Aging Center, Department of Neurology, UCSF, San Francisco, California, USA.; 9 Pathology Service, San Francisco Veterans Health Care System, San Francisco, California, USA.

**Keywords:** Neuroscience, Dementia, Molecular pathology, Neurodegeneration

## Abstract

Mutations in the human progranulin (*GRN*) gene are a leading cause of frontotemporal lobar degeneration (FTLD). While previous studies implicate aberrant microglial activation as a disease-driving factor in neurodegeneration in the thalamocortical circuit in *Grn^–/–^* mice, the exact mechanism for neurodegeneration in FTLD-*GRN* remains unclear. By performing comparative single-cell transcriptomics in the thalamus and frontal cortex of *Grn^–/–^* mice and patients with FTLD-*GRN*, we have uncovered a highly conserved astroglial pathology characterized by upregulation of gap junction protein GJA1, water channel AQP4, and lipid-binding protein APOE, and downregulation of glutamate transporter SLC1A2 that promoted profound synaptic degeneration across the two species. This astroglial toxicity could be recapitulated in mouse astrocyte-neuron cocultures and by transplanting induced pluripotent stem cell–derived astrocytes to cortical organoids, where progranulin-deficient astrocytes promoted synaptic degeneration, neuronal stress, and TDP-43 proteinopathy. Together, these results reveal a previously unappreciated astroglial pathology as a potential key mechanism in neurodegeneration in FTLD-*GRN*.

## Graphical abstract

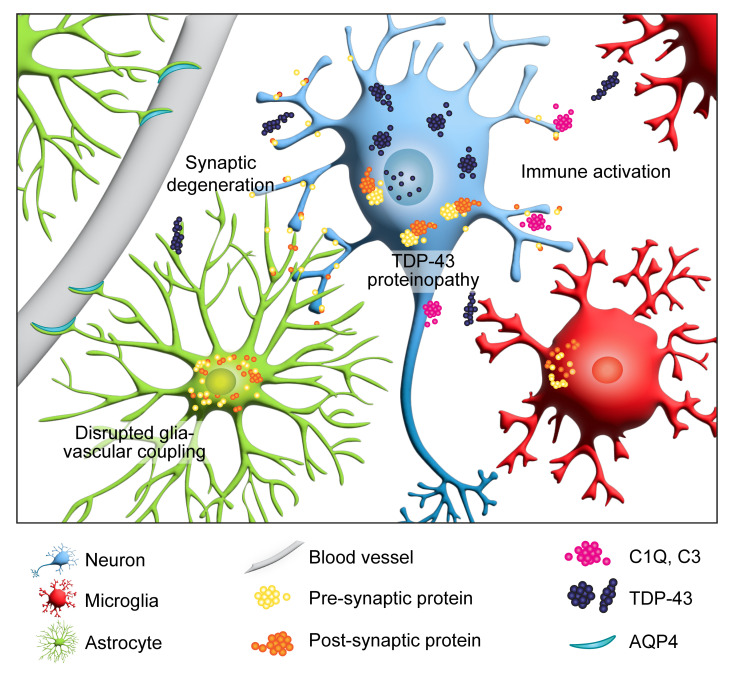

## Introduction

Frontotemporal dementia (FTD) is a common neurodegenerative disease in patients under 65 years of age. The clinical manifestations of FTD include progressive behavioral changes and deterioration in language skills and motor dysfunction, whereas the neuropathological features — collectively known as frontotemporal lobar degeneration (FTLD) — show severe atrophy in the frontal, temporal, and insular cortex, as well as striatum and thalamus (1). Dominant mutations in the Progranulin (*GRN*) gene are one leading cause for FTLD; these mutations activate nonsense-mediated decay of *GRN* mRNA and haploinsufficiency in progranulin (PGRN) protein levels (2, 3). To model how PGRN deficiency promotes neurodegeneration, several studies have shown that mice with a homozygous deletion in *Grn* (*Grn^–/–^*) or 2 copies of humanized *Grn^R493X^* allele exhibit age-dependent microglial activation, which promotes excessive synaptic pruning in the thalamocortical circuit and premature death (4, 5). Furthermore, single-nuclei RNA-Seq (snRNA-Seq) using micro-dissected thalami from an aging cohort of *Grn^+/+^* and *Grn^–/–^* mice show that persistent activation of *Grn^–/–^* microglia promotes neuronal cell death and TDP-43 proteinopathy during brain aging (6). 

There are several intriguing characteristics associated with the neuroinflammation in *Grn^–/–^* mice. First, the aberrant activation of microglia in *Grn^–/–^* mice appears to be neural circuit-specific, which preferentially affects the thalamus and is less prominent in the cerebral cortex (4, 7, 8). Second, microglia in the thalamus of *Grn^–/–^* mice undergo transcriptomic changes that age-dependently transition microglia from a homeostatic state to a disease-associated state, which promotes synaptic pruning followed by loss of excitatory neurons during end-stage disease (6). Finally, a recent functional MRI (fMRI) study shows that presymptomatic *GRN* mutation carriers exhibit hyperconnectivity within 4 functional thalamocortical networks (9). These results suggest that microglial toxicity is a potential disease-driving factor that promotes neurodegeneration in the thalamocortical circuit in both humans and mice with PGRN deficiency.

Several studies indicate that neuroinflammation may contribute to the pathogenesis of FTLD-*GRN*. For instance, a systems biology approach to investigate FTLD caused by mutations in *MAPT* or *GRN* shows that neurodegeneration-associated inflammation (NAI) module is significantly upregulated in FTLD-*GRN* cases, similar to 18-month-old *Grn^–/–^* brains (4, 10). In addition, this study identifies another disease-associated gene module in patients with FTLD-*GRN*, called the neurodegeneration-associated synaptic (NAS) module, which is characterized by the downregulation of hub genes involved in axon guidance, synaptic organization, and plasticity. Despite these intriguing results, the exact role of neuroinflammation in neurodegeneration in FTLD-*GRN* remains poorly understood.

## Results

### Parallel glial pathology in Grn^–/–^ and FTLD-GRN brains.

**Figure 1:**
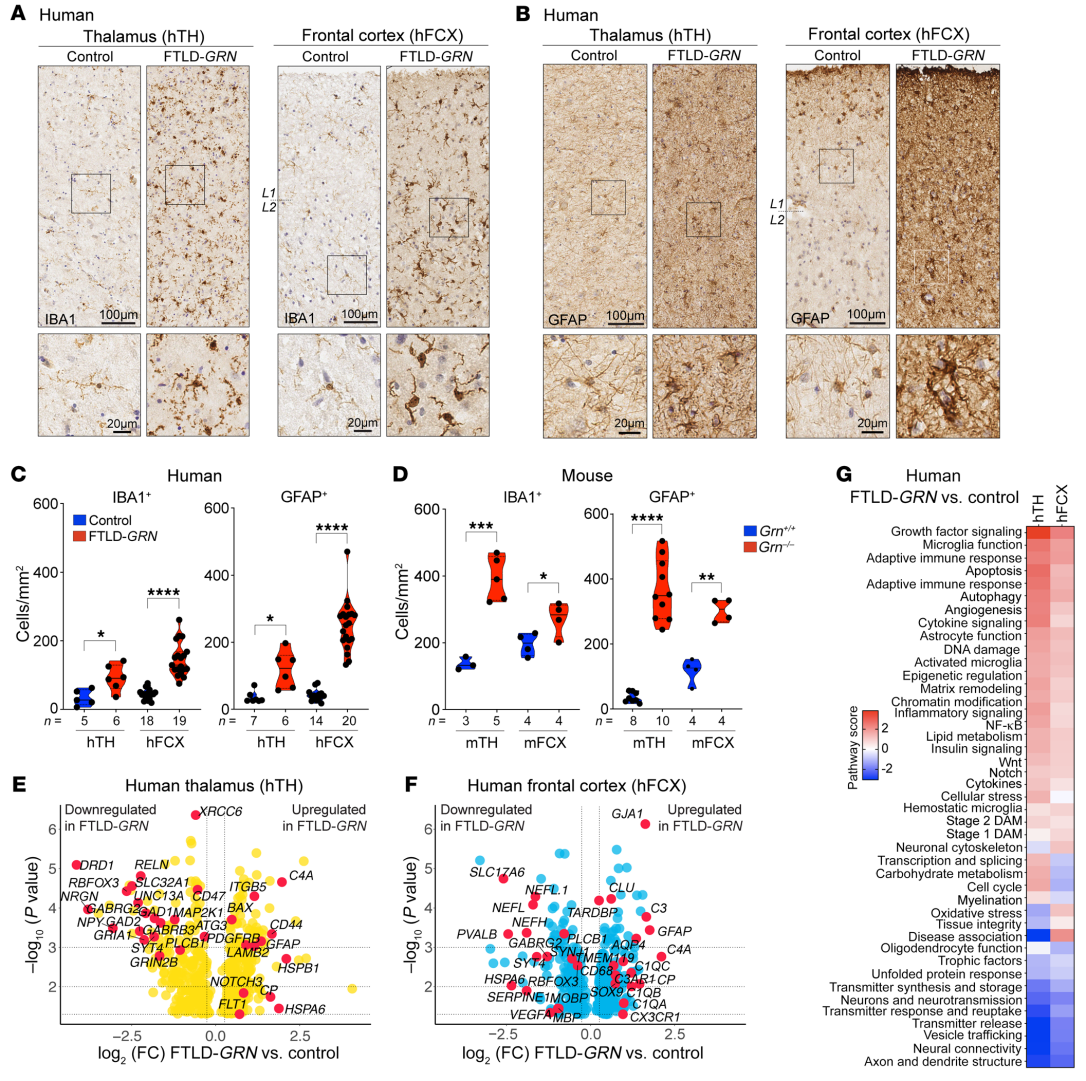
Glial pathology in thalamus and frontal cortex of patients with FTLD-*GRN* and *Grn^–/–^* mice. (**A** and **B**) Immunostains for IBA1 and GFAP in the thalamus (hTH) and frontal cortex (hFCX) in control and FTLD-*GRN* cases. (**C** and **D**) Quantification of IBA1^+^ microglia and GFAP^+^ astrocyte density in hTH and hFCX (**C**) and mFCX and mTH (**D**). Statistics for IBA1^+^ microglia in hTH, hFCX, mTH, and mFCX use parametric 2-tailed Student’s *t* test and for GFAP^+^ astrocytes parametric (for hFCX, mTH, and mFCX) or nonparametric 2-tailed Student’s *t* test (for hTH). (**E** and **F**) Volcano plots showing DEGs in hTH (**E**) and hFCX (**F**) from the Nanostring nCounter neuropathology and neuroinflammation panels. (**G**) Heatmap showing nCounter pathway scores in hTH and hFCX of patients with FTLD-*GRN*. L1/2 indicates the border between layers 1 and 2 of the frontal cortex. All data represent mean ± SEM, and *n* represents the number of independent human and mouse samples used. *P < 0.05, **P < 0.01 ***P < 0.001, ****P < 0.0001. Scale bars: 100 μm; 20 μm (inset).

To investigate the impacts of *GRN* mutations in the thalamocortical circuit in people and mice, we first compared the glial pathology in the thalamus and frontal cortex in patients with FTLD-*GRN* and 19-month-old *Grn^–/–^* mice. We chose 19-month-old mice because this was close to the median survival of *Grn^–/–^* mice (4, 5). Using immunostains for Ionized Calcium-Binding Adapter Molecule-1 (IBA1) and glial fibrillary acidic protein (GFAP), we found significant increases in microglia and astrocytes in the human thalamus (hTH) and human frontal cortex (hFCX) of patients with FTLD-*GRN* (Figure 1, A–C). Similar to FTLD-*GRN* brains, 19-month-old *Grn^–/–^* mouse brains also showed increases in IBA1^+^ microglia and GFAP^+^ astrocytes in the mouse thalamus (mTH) and in the sensorimotor cortex of the mouse frontal lobe (mFCX) (Figure 1D). Interestingly, side-by-side comparisons in patients with FTLD-*GRN* and 19-month-old *Grn^–/–^* mice showed that the microglial and astroglial density in patients with FTLD-*GRN* was higher in hFCX than in hTH (Figure 1C), whereas microglial and astroglial density in 19-month-old *Grn^–/–^* mice was higher in mTH than mFCX (Figure 1D).

**Figure 2:**
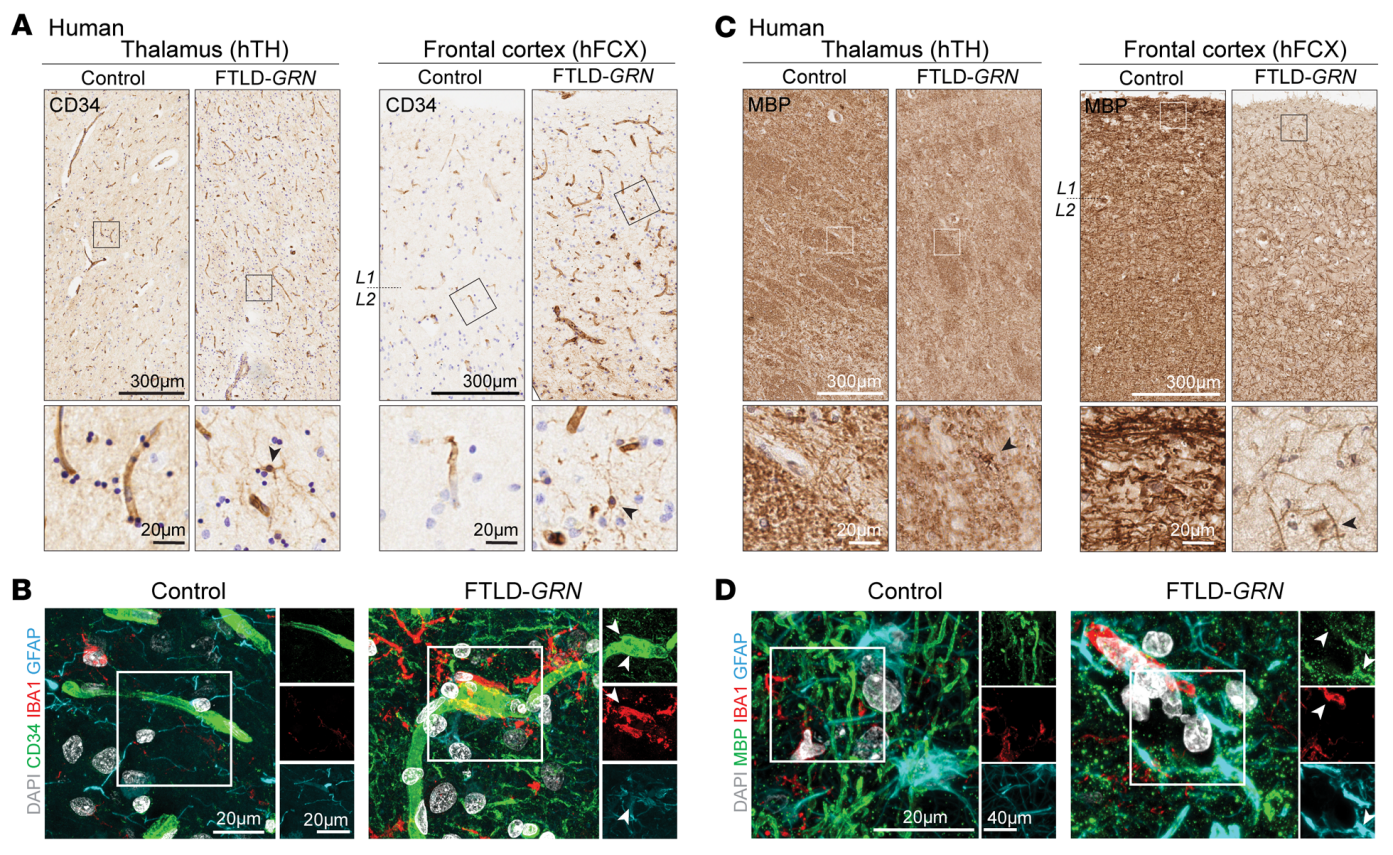
Effect of glial pathology on the vasculature and myelination in thalamus and frontal cortex of patients with FTLD-*GRN*. (**A**) Immunostains for CD34 in hTH and hFCX in control and FTLD-*GRN* cases. (**B**) Confocal images for CD34, IBA1, and GFAP showing IBA1^+^ microglia engulfing CD34^+^ debris in FTLD-*GRN* (arrowheads). (**C**) Immunohistochemical stains for MBP in hTH and hFCX in control and FTLD-*GRN* cases. (**D**) Confocal images showing IBA1^+^ microglia and GFAP^+^ astrocytes engulfing MBP^+^ myelin debris (arrowheads) in FTLD-*GRN*. L1/2 indicates the border between layers 1 and 2 of the frontal cortex. Scale bars: 20 μm (**A** and **C**, bottom; **B**; and **D**, left); 40 μm (**D**, right); 300 μm (**A** and **C**, top).

Next, we used the Nanostring nCounter platform to analyze 770 neuroinflammation-related and 770 neuropathology-related genes in hTH and hFCX from 11 individuals without FTLD-*GRN* and 10 patients with FTLD-*GRN* (Supplemental Table 1; supplemental material available online with this article; https://doi.org/10.1172/JCI164919DS1). This analysis showed that the gene expression profiles in hTH and hFCX were distinctly different in individuals with FTLD-*GRN,* based on the large number of differentially expressed genes (DEGs). As highlighted by the volcano plots for the combined neuroinflammation and neuropathology panels in hTH and hFCX, the upregulated DEGs included *TYROBP*, *FCGR3A*, *TLR4*, *CD68*, *C1QA*, *C1QB*, *C1QC*, *C3*, *C3AR1*, *C4A*, *CX3CR1*, *TMEM119*, *GFAP*, *AQP4*, *GJA1*, *CLU*, *PDGFRB*, *FLT1* (VEGFR1), *NOTCH3*, and *TGFBR2*. The downregulated genes included *GAD1*, *GAD2*, *NPY*, *PVALB*, *GABRG2*, *RELN*, *RBFOX3*, *NEFL*, *NEFH*, *UNC13A*, *MBP*, and *MOBP* (Figure 1, E and F, Supplemental Table 2). Functional pathways defined by these DEGs revealed upregulation of the innate and adaptive immune responses, complement activation, microglia and astrocyte functions, cytokine signaling, angiogenesis, and downregulation of neurotransmission, axon/dendrite structure, and oligodendroglia function (Figure 1G). Consistent with the observed transcriptomic changes, immunostains using endothelial cell marker CD34 highlighted the delicate vasculature in hTH and hFCX from control individuals. However, the vasculatures in the same brain regions in patients with FTLD-*GRN* showed abundant CD34-containing glial cells (Figure 2A, arrowheads in bottom panels). Confocal microscopy showed that much CD34^+^ debris was inside IBA1^+^ microglia and GFAP^+^ astrocytes (Figure 2B, arrowheads). Similarly, immunostains for MBP showed reduced myelinated axons in hTH and hFCX in individuals with FTLD-*GRN* (Figure 2C), with much myelin debris engulfed by IBA1^+^ microglia and GFAP^+^ astrocytes (Figure 2D, arrowheads).

### snRNA-Seq in the thalamocortical circuit in FTLD-GRN and Grn^–/–^ brain.

**Figure 3:**
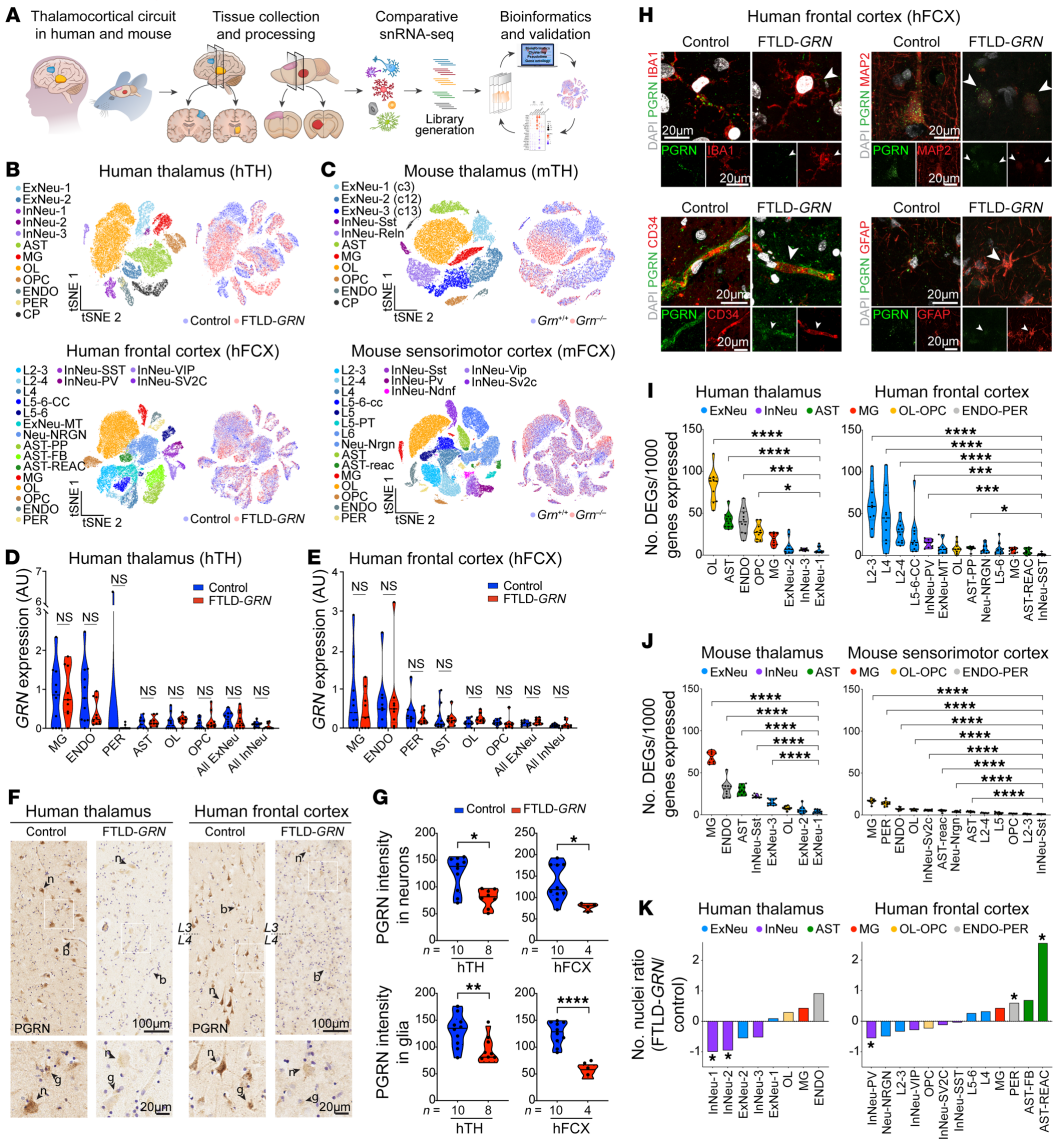
snRNA-Seq analyses in the thalamus and frontal cortex of individuals with and without FTLD-*GRN*, and *Grn^+/+^* and *Grn^–/–^* mice. (**A**) A schematic diagram for single-cell transcriptomics using the thalamus and frontal cortex from control and FTLD-*GRN* cases and 19-month-old *Grn^+/+^* and *Grn^–/–^* mice. (**B** and **C**) *t*SNE plots highlighting the distribution of cell clusters from hTH, hFCX from control and FTLD-*GRN* cases (**B**), mTH and mFCX from 19-month-old *Grn^+/+^* and *Grn^–/–^* mice (**C**). (**D** and **E**) Violin plots showing *GRN* RNA levels in all the cell types in hTH and hFCX from individuals with and without FTLD-*GRN*. Statistics uses multiple unpaired Student’s 2-tailed *t* tests. (**F**) Immunostains for PGRN in hTH and hFCX of patients with FTLD-*GRN*. n, neuron; b, blood vessel; g, glia cell; L3, layer 3; L4, layer 4. Scale bars: 100 μm; 20 μm (inset). (**G**) Quttantification of PGRN intensity in neurons and glia in hTH and hFCX in individuals with and without FTLD-*GRN*. Statistics uses 2-tailed Student’s *t* test, n represents the number of independent samples used. (**H**) Confocal images of PGRN in IBA1^+^ microglia, CD34^+^ endothelial cells, MAP2^+^ neurons, and GFAP^+^ astrocytes, in the hFCX of control and FTLD-*GRN* patients. Scale bars: 20 μm. (**I** and **J**) Gene burden score for each cell cluster in the hTH, hFCX, mTH, and mFCX. Statistics use parametric (mTH, mFCX) or nonparametric (hTH, hFCX) 1-way ANOVA. (**K**) Bar graphs showing the ratio of the number of nuclei captured in the hTH and hFCX in control and FTLD-*GRN* patients. Statistics use nonparametric 2-tailed Student’s *t* test (hTH: ExNeu-1, ExNeu-2, and MG; hFCX: AST-REAC) or parametric 2-tailed Student’s *t* test (for remaining clusters). ExNeu, excitatiry neurons; InNeu, inhibitory neurons; AST, astrocytes; MG, microglia; OL, oligodendroglia; OPC, oligodendroglial precursors; ENDO, endothelial cells; PER, pericytes. All data represent mean ± SEM. *P < 0.05, **P < 0.01, ***P < 0.001, ****P < 0.0001. NS, not significant.

To investigate the interplays between glial and neuronal pathology caused by *GRN* mutations, we performed snRNA-Seq analysis using postmortem tissues from hTH and hFCX from 10 patients with FTLD-*GRN* and 11 individuals without FTLD-*GRN* as age-matched controls (Figure 3A and Supplemental Figures 1 and 2). In parallel, we performed snRNA-Seq using micro-dissected mFCX of 19-month-old *Grn^+/+^* and *Grn^–/–^* mice (Figure 3A and Supplemental Figure 3) and leveraged the previously published snRNA-Seq data sets from the mTH of *Grn^+/+^* and *Grn^–/–^* mice at the same age (6). Clustering using known cell type-specific markers identified 12 and 19 distinct cell clusters in hTH and hFCX, respectively (Figure 3B). The cell clusters in hTH included 2 excitatory neuron clusters (ExNeu-1 [NTNG1^+^;TUBA1B^+^] and ExNeu-2 [NTNG1^–^;TUBA1B^+^]), 3 inhibitory neuron clusters (InNeu-1 [BCL11B^+^;LAMP5^+^], InNeu-2 [BCL11B^+^;LAMP5^–^], and InNeu-3 [NR2F2^+^]), astrocytes (AST), microglia (MG), oligodendroglia (OL), oligodendroglial precursors (OPC), endothelial cells (ENDO), and pericytes (PER) (Supplemental Figure 1). In contrast, cell clusters in hFCX encompassed layer-specific excitatory neurons (L2-3, L2-4, L4, L5-6-CC, and L5-6), nonlayer-specific excitatory neurons expressing Neurogranin (ExNeu-NRGN), excitatory neurons with enriched mitochondrial genes (ExNeu-MT), 4 inhibitory neuron clusters (InNeu-SST, InNeu-PV, InNeu-VIP, and InNeu-SV2C), 3 astroglial clusters (protoplasmic astrocytes [AST-PP], fibrous astrocytes [AST-FB], and reactive astrocytes [AST-REAC]), MG, OL, OPC, ENDO, and PER (Supplemental Figure 2) (11, 12). Finally, clustering in 19-month-old *Grn^+/+^* and *Grn^–/–^* mTH and mFCX revealed 11 clusters and 20 clusters, respectively (Figure 3C and Supplemental Figure 3).

To characterize the microglial transcriptomes in the hTH and hFCX, we compared several key homeostatic microglial genes and subclustering analyses between control and FTLD-*GRN* cases (Supplemental Figure 4, A–D). In addition, we compared human microglial transcriptomes from our data sets with peripheral immune cells (13) and found remarkable similarity with those in macrophages (Supplemental Figure 4, E–H). For the astroglial clusters, we generated a number of violin plots to compare the expression of key astroglial genes in individuals with and without FTLD-*GRN* and performed immunostains using ALDH1L1 ab to further characterize astroglial phenotypes in the hTH and hFCX in control and FTLD-*GRN* cases (Supplemental Figure 5). Next, we examined *GRN* RNA levels in each cell cluster in the hTH and hFCX. Similar to the snRNA-Seq data from *Grn^+/+^* mice (6), the MG clusters in hTH and hFCX in individuals with and without FTLD-*GRN* showed similar levels of *GRN* RNA — the most abundant levels among the clusters — followed by the ENDO and PER clusters (Figure 3, D and E). All other cell clusters showed relatively modest *GRN* RNA expression. Interestingly, although *GRN* RNA was detected in the hTH and hFCX of patients with FTLD-*GRN*, because these were nuclear transcripts not subject to nonsense-mediated decay in the cytoplasm (14), immunostains using PGRN-specific abs showed a marked reduction of PGRN proteins (Figure 3, F–H), supporting a drastic loss of PGRN in the brains of patients with FTLD-*GRN* at the end stage of the disease (2, 3).

To characterize the effects of *GRN* mutations, we calculated the gene burden scores in the hTH and hFCX in patients with FTLD-*GRN* and in the mTH and mFCX in 19-month-old *Grn^–/–^* mice (6, 12). These results showed that, similar to mTH in *Grn^–/–^* mice (6), the nonneuronal cell clusters in hTH of patients with FTLD-*GRN*, including OL, AST, ENDO, OPC, and MG, also showed significantly higher gene burden scores than the neuronal clusters (Figure 3I, left panel). Conversely, gene burden scores in hFCX showed much more significant changes in the neuronal clusters — especially those in L2-3, L4, L2-4 and L5-6-CC — than those in the nonneuronal clusters, including OL, AST-PP, MG, and AST-REAC (Figure 3I, right panel). Compared with mTH, mFCX in 19-month-old *Grn^–/–^* mice showed very modest increases in gene burden scores in all clusters (Figure 3J). Quantification of the number of nuclei for each cluster in hTH and hFCX in individuals with FTLD-*GRN* revealed losses of InNeu-1 and InNeu-2, and modest losses in ExNeu-2 and InNeu-3 clusters in hTH in individuals with FTLD-*GRN*. In addition, hTH in patients with FTLD-*GRN* showed modest increases in nuclei counts in nonneuronal cells (Figure 3K, left panel). Similar analyses revealed a significant loss of InNeu-PV and a marked increase of AST-REAC in hFCX in individuals with FTLD-*GRN* (Figure 3K, right panel), but no significant change in the clusters in mFCX (Supplemental Figure 3I).

### Shared transcriptomic changes in Grn^–/–^ and FTLD-GRN microglia in the thalamus.

**Figure 4:**
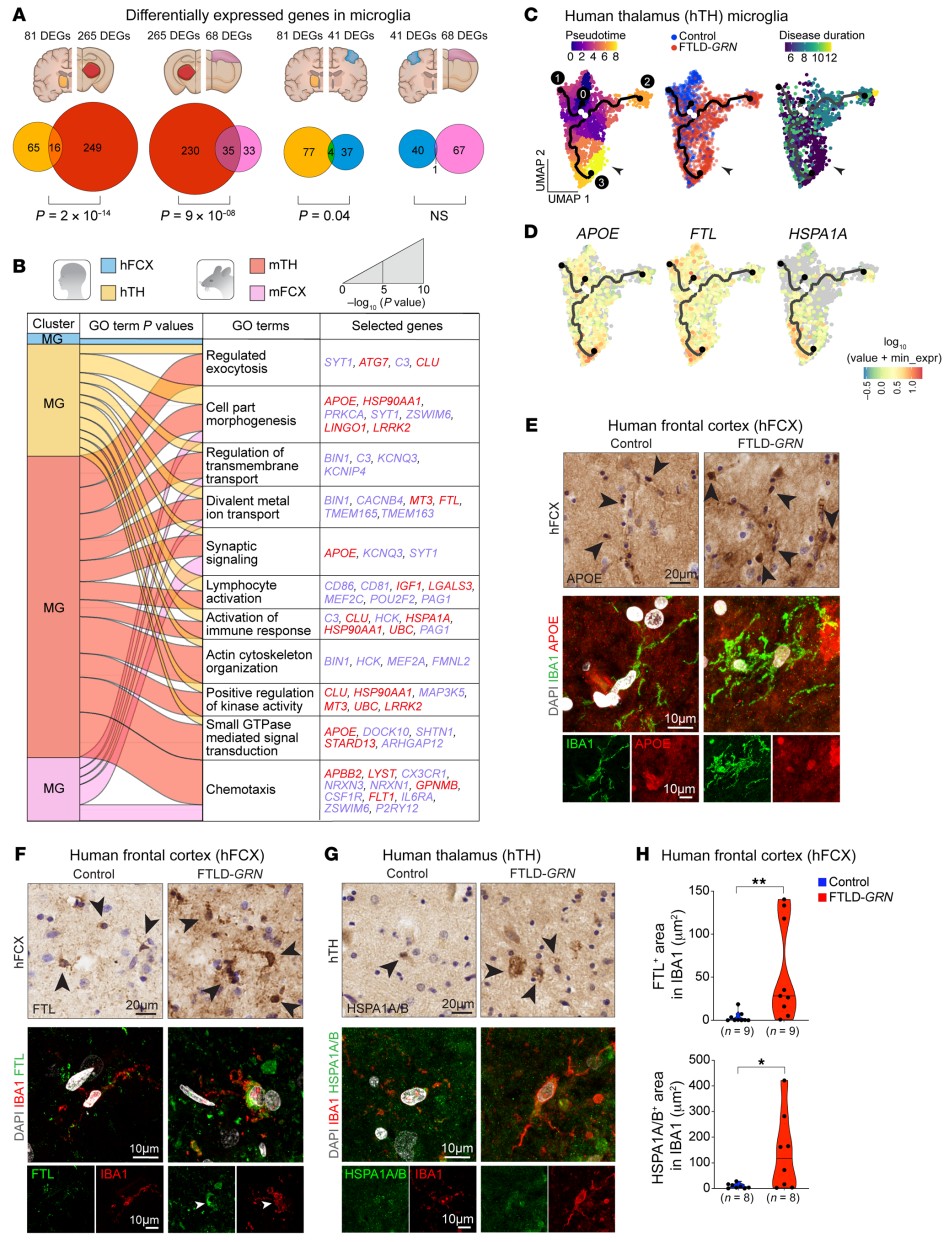
Transcriptomic and phenotypic characterizations of microglia in patients with FTLD-*GRN* and 19-month-old *Grn^–/–^* mice. (**A**) Venn diagrams show the overlap in the DEGs in the microglial (MG) clusters from hTH, hFCX, mTH, and mFCX. Statistics uses hypergeometric tests. (**B**) Alluvial plot showing the overlap of the GO terms defined by DEGs from the MG clusters in hTH, hFCX, mTH, and mFCX. The width of the band for each GO terms represent its *P* value calculated based on the cumulative hypergeometric distribution. Blue and red gene names indicate down- and up-regulated DEGs, respectively. (**C**) Pseudotime analysis of the MG clusters in hTH based on UMAP (left panel), individuals with FTLD-*GRN* or without (control), or disease duration (right panel). (**D**) Expression levels of *APOE*, *FTL*, and *HSPA1A* projected onto the UMAP for the MG cluster in hTH. Scale represents log_10_ of the expression of representative genes. (**E**–**G**) Immunostains and confocal images showing the expression of APOE, FTL, and HSPA1A/B in IBA1^+^ microglia in control and FTLD-*GRN* cases. (**H**) Quantification of FTL and HSPA1A/B signal intensity on immunostains. Statistics use 2-tailed Student’s *t* test, and *n* represents the number of independent samples tested. All data represent mean ± SEM. *P < 0.05, **P < 0.01. Scale bars: 20μm (immunostains); 10 μm (confocal images).

Since the nCounter results showed robust changes in gene expression related to innate and adaptive immune responses, we used snRNA-Seq analysis to identify species- and region-specific changes in the microglial phenotypes in hTH and hFCX in FTLD-*GRN* cases. First, we compared the DEGs in the MG clusters in hTH and hFCX with those in mTH and mFCX in 19-month-old *Grn^–/–^* mice. This revealed significant overlaps of the DEGs in microglia from hTH and mTH, and in microglia from mTH and mFCX. In contrast, microglia from hTH and hFCX shared a limited number of DEGs, while microglia from hFCX and mFCX showed no significant overlap of DEGs (Figure 4A). Next, we used alluvial plots and heatmaps to compare the top 20 gene ontology (GO) terms defined by the DEGs in the MG clusters in hTH, hFCX, mTH, and mFCX (Figure 4B, Supplemental Figure 6A, and Supplemental Tables 3 and 4). These results showed that the MG clusters in hTH and mTH shared an extensive list of GO terms defined by many shared DEGs, including those related to chemotaxis (*APBB2*, *GPNMB*, and *LYST*), activation of immune response (*C3*, *CLU*, and *HSPA1A*), regulated exocytosis (*SYT1*, *ATG7*, *C3*, and *CLU*), cell part morphogenesis (*APOE*, *HSP90AA1*, and *LINGO1*), regulation of transmembrane transport and synaptic signaling (*BIN1* and *KCNQ3*), divalent metal ion transport (*MT3*, *FTL*, *TMEM163*, and *TMEM165*), lymphocyte activation (*CD86*, *CD81*, *IGF1*, *LGALS3*, and *MEF2C*), actin cytoskeleton organization (*BIN1*, *HCK*, *MEF2A*, and *FMNL2*), positive regulation of kinase activity (*MAP3K5* and *LRRK2*), and small GTPase mediated signal transduction (*STARD13* and *ARHGAP12*) (Figure 4B and Supplemental Figure 6A). In contrast, the GO terms defined by the MG DEGs in hFCX were distinctly different from those from mFCX and showed very limited overlap with those from hTH, sharing only 1 GO term.

To further illuminate the underlying biological processes in disease microglia, we performed unsupervised trajectory and pseudotime analysis to determine whether changes in gene expression in microglia in patients with FTLD-*GRN* correlated with disease (15, 16). This analysis showed that microglia from hTH in individuals with and without FTLD*-GRN* exhibited 3 distinct trajectories. Trajectory 1 contained microglia predominantly from control hTH with a shorter pseudotime scale, while trajectories 2 and 3 were more enriched with microglia from hTH in individuals with FTLD-*GRN* and had longer pseudotime scales (Figure 4C). Next, we projected the disease duration in each individual with FTLD-*GRN* onto the pseudotime map and showed that microglia from individuals with FTLD-*GRN* in trajectory 2 had a mixed disease duration, whereas microglia in trajectory 3 had much shorter disease duration, usually of less than 6 years (Figure 4C). Several DEGs in the MG clusters in FTLD-*GRN* hTH showed more enrichments that correlated with trajectories 2 and 3, including *APOE*, *FTL*, *LINGO1*, and *HSPA1A*, and complements *C1QA*, *C1QB*, and *C1QC* (Figure 4D and Supplemental Figure 6B). In contrast, pseudotime analysis for microglia in hFCX showed no correlation of trajectory that separated microglia from the control and FTLD-*GRN* samples (Supplemental Figure 6C). Consistent with the transcriptomic data, immunostains showed upregulation of for FTL, HSPA1A/B, CLU, and C1q, and downregulation of KCNQ3 in microglia in hFCX and hTH in patients with FTLD-*GRN* (Figure 4, E–H and Supplemental Figure 6, D–G). Finally, we compared the microglial transcriptomes from hTH and hFCX from individuals with FTLD-*GRN* with those from patients with Alzheimer’s disease (AD) (17–19). These results showed that microglia from hTH in individuals with FTLD-*GRN* shared more GO terms with those from patients with AD than with those in hFCX from patients with FTLD-*GRN* (Supplemental Figure 6, H and I).

### Conserved transcriptomic changes in FTLD-GRN and Grn^–/–^ astrocytes.

**Figure 5:**
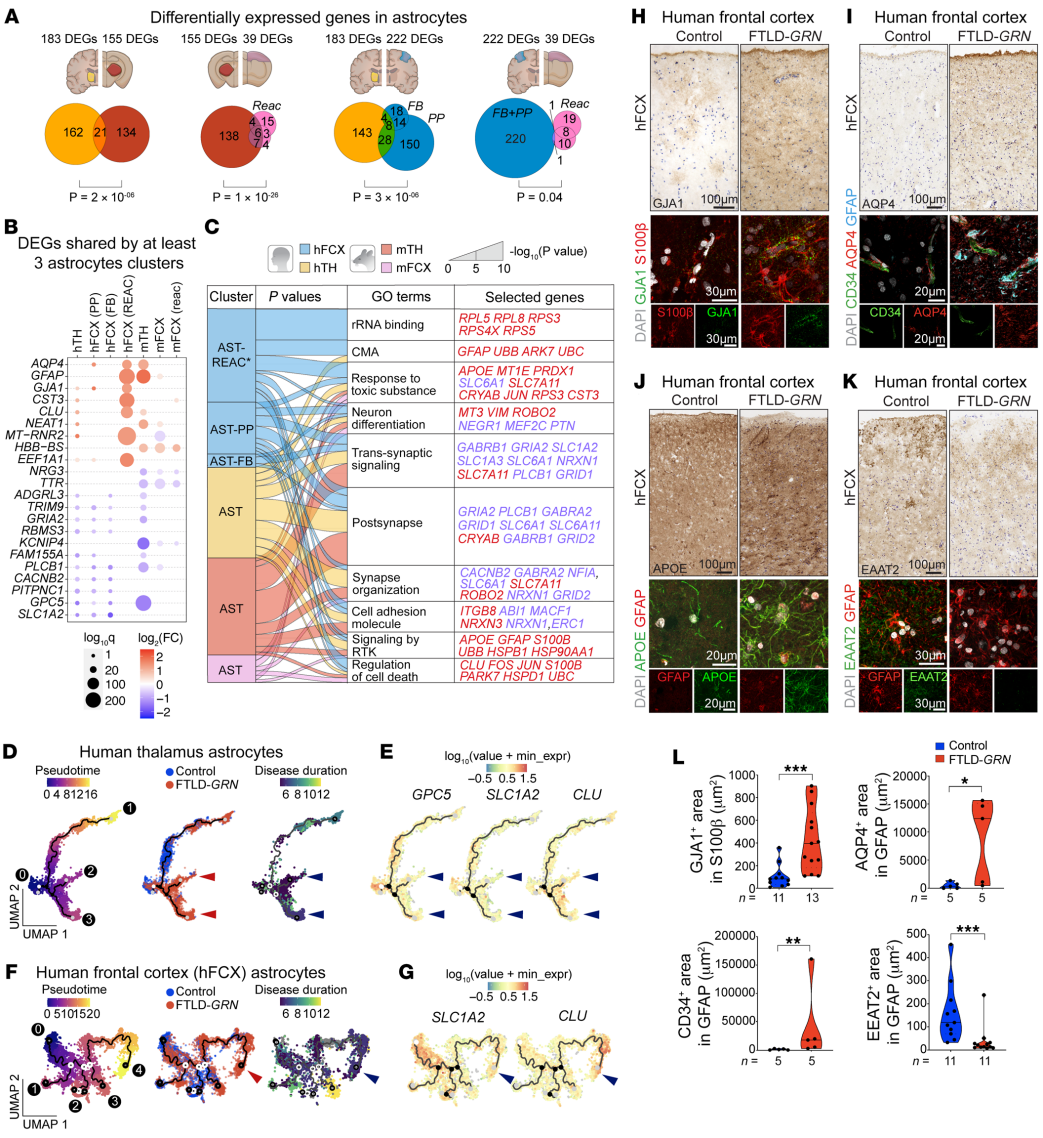
Conserved transcriptomic and phenotypic changes in astrocytes in FTLD-*GRN* and *Grn^–/–^* mice. (**A**) Venn diagrams showing the overlap of DEGs in the astroglial (AST) clusters from hTH, hFCX, mTH and mFCX. Statistics uses hypergeometric tests. (**B**) DEGs shared by ≥3 AST clusters in mTH, mFCX, hTH and hFCX, with a scale indicating log_2_ Fold Change that has a cutoff from –0.5 to 0.5. Dot size indicates the log_10_ Q value for each DEG and color scale indicates log_2_ Fold Change. Blue and red indicate down- and up-regulated DEGs, respectively. (**C**) Alluvial plot showing the overlap of GO terms defined by DEGs from AST clusters in hTH, hFCX, mTH, and mFCX. Band width represents *P* values calculated by accumulative hypergeometric distribution. CMA, chaperone-mediated autophagy; RKT, receptor tyrosine kinases. (**D**) Pseudotime analysis of AST clusters in hTH based on UMAP (left panel), control versus. FTLD-*GRN* (middle panel), or disease duration (right panel). (**E**) Expression of *GPC5*, *SLC1A2*, and *CLU* projected onto UMAP of AST cluster in hTH. (**F**) Pseudotime analysis of the AST clusters in hFCX based on UMAP (left panel), control versus FTLD-*GRN* (middle panel), or disease duration (right panel). (**G**) Expression of *SLC1A2* and *CLU* projected onto UMAP of AST cluster in hFCX. (**H**–**K**) Immunohistochemical (top) and confocal images (bottom) of GJA1 (**H**), AQP4 (**I**), APOE (**J**), and EAAT2 (**K**) in hFCX of control and FTLD-*GRN*. (**L**) Quantification of GJA1, CD34, AQP4, and EAAT2 expression on immunostains in control and FTLD-*GRN* cases. Statistics uses 2-tailed Student’s *t* test, and *n* represents the number of independent samples tested. All data represent mean ± SEM. *P < 0.05, **P < 0.01, ***P < 0.005. Scale bars: 100μm (immunostains); 20 μm and 30 μm (confocal images).

Compared with the MG clusters, the AST clusters in mTH, mFCX, and hTH shared significantly larger number of DEGs than the AST clusters in hFCX and mFCX (Figure 5, A and B and Supplemental Figure 7A). In addition, alluvial plots showed extensive overlap among the GO terms defined by the DEGs in the AST clusters in hTH and hFCX in FTLD-*GRN* samples and mTH in *Grn^–/–^* mouse brain samples (Figure 5C). Although AST clusters from mFCX in *Grn^–/–^* mouse brain samples shared limited DEGs with those from mTH and hTH and hFCX in FTLD-*GRN* samples, the GO terms defined by astroglial DEGs in mFCX showed significant overlap with those from other regions (Figure 5C and Supplemental Figure 7B). Collectively, the GO terms shared by most AST clusters in hTH, hFCX, mTH, and mFCX included many upregulated genes related to response to toxic substances (*MBP*, *MT1E*, and *PRDX1*), signaling by receptor tyrosine kinase (*PDGFRB*, *S100B*, *UBB*, and *UBC*), and chaperone-mediated autophagy (*GFAP*, *EEF1A1*, and *PARK7*), and down-regulated genes that were implicated in recycling of neurotransmitters, transsynaptic signaling, and synapse organization (*SLC1A2* (*EAAT2*), *SLC1A3* (*EAAT1*), *SLC6A1*, *GRIA2*, *NRXN1*, and *PLCB1*). Comparisons among all AST clusters in the brains of *Grn*^–/–^ mice and individuals with FTLD-*GRN* revealed several astroglial genes that were consistently upregulated (*AQP4*, *GFAP*, *CST3*, and *CLU*) or downregulated (*GRIA2*, *PLCB1*, and *GPC5*) (Figure 5B, Supplemental Figure 7A, and Supplemental Table 3).

Next, we performed unsupervised trajectory and pseudotime analysis to identify disease-specific progression of astrocytes in FTLD-*GRN* cases. Like the MG cluster in hTH, we also identified 3 trajectories for hTH AST clusters in control and FTLD-*GRN* cases (Figure 5D, arrowheads). Trajectory 1 was shared between control and FTLD-*GRN*, whereas trajectories 2 and 3 were more enriched with AST clusters from individuals with FTLD-*GRN* and were associated with shorter disease duration and upregulation of *GFAP*, *GJA1*, *APOE*, and *CLU*, and downregulation of *GPC5* and *SLC1A2* (Figure 5E and Supplemental Figure 7C). Similar analysis in AST clusters from hFCX in patients with FTLD-*GRN* revealed 4 distinct trajectories. Trajectories 1–3 were shared between individuals with and without FTLD-*GRN*, whereas trajectory 4 showed enriched representation of astrocytes from patients with FTLD-*GRN* and correlated with shorter disease duration. Similar to trajectory 3 of the astrocytes in hTH in patients with FTLD-*GRN*, trajectory 4 in hFCX in patients with FTLD-*GRN* showed upregulation of *GFAP*, *AQP4*, *APOE*, and *CLU*, and downregulation of *SLC1A2* (Figure 5, F and G and Supplemental Figure 7D).

To validate the transcriptomic changes in the AST clusters, we performed immunostains to characterize the astroglial phenotypes in the hTH and hFCX in patients with FTLD-*GRN*. In control hFCX, astrocytes expressed gap junction protein GJA1 (Connexin 43) that decorated the thin, delicate astroglial processes in layers 1–3. In contrast, the same layers in hFCX in patients with FTLD-*GRN* showed intense and diffuse GJA1 expression in almost all astrocytes (Figure 5H). Another important astroglial protein investigated was the selective water channel AQP4, which, like GJA1, was detected exclusively in GFAP^+^ astroglial processes touching CD34^+^ vasculature (Figure 5I), consistent with the reported role of AQP4 in regulating water uptake at the blood-brain barrier via astroglial endfeet (20, 21). In contrast, hFCX in individuals with FTLD-*GRN* showed many markedly enlarged GFAP^+^ astrocytic processes, which contained abundant AQP4 proteins, encasing the blood vessels and in numerous GFAP^+^ astrocytic processes in the neuropil and surrounding the blood vessels. These resembled the phenotypes in transgenic mice where overexpression of AQP4 increased water intake and cerebral edema (22). In addition, astrocytes in hFCX of individuals with FTLD-*GRN* showed a marked upregulation of S100β and APOE (Figure 5, H and J). Interestingly, upregulation of GJA1 and APOE have been identified as integral parts of AD-coexpression network in astrocytes (23, 24). In addition, astrocytes in hFCX of individuals with FTLD-*GRN* showed downregulation of SLC1A2 (EAAT2), SLC1A3 (EAAT1), GPC5 (Glypican 5), and AMPA receptor subunits GRIA2/4 (Figure 5K and L, Supplemental Figure 7, E–G). Interestingly, downregulation of these genes, with the exception of *GPC5*, has been associated with brain aging in mice (25), whereas downregulation of EAAT2 can increase excitotoxicity in Huntington’s disease and amyotrophic lateral sclerosis models (26–28). Finally, compared with microglia, the astroglial clusters from hTH and hFCX in individuals with FTLD-*GRN* shared more DEGs and GO terms with astrocytes from individuals with AD (Supplemental Figure 7, L and M).

### Synaptic and dendritic degeneration in FTLD-GRN neurons.

**Figure 6:**
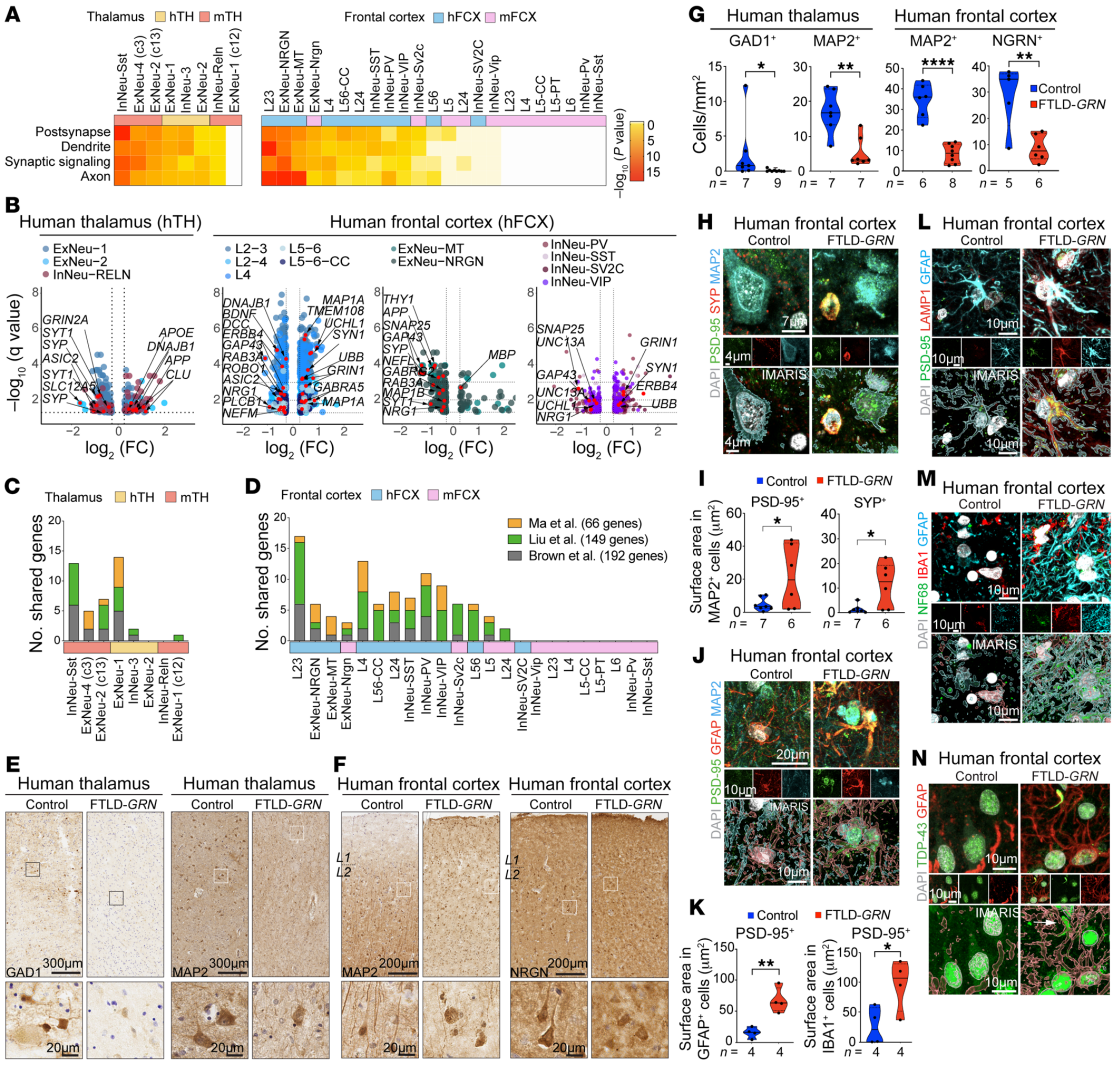
Neuronal phenotypes in patients with FTLD-*GRN* and *Grn^–/–^* mice. (**A**) Heatmaps showing the top 4 GO terms shared by the neuronal clusters in hTH, hFCX, mTH, and mFCX. Statistics use hypergeometric tests. (**B**) Volcano plots showing DEGs in neuronal clusters in hTH and hFCX and genes related to the GO terms in **A**. (**C** and **D**) Bar graphs showing the number of DEGs in the neuronal clusters in patients with FTLD-*GRN* and *Grn^–/–^* mice that overlap with TDP-43-mediated misspliced genes. (**E** and **F**) Immunostains for GAD1 and MAP2 in hTH (**E**) and for MAP2 and NRGN in hFCX (**F**) in control and FTLD-*GRN* cases. Scale bars: 300 μm (**E**, top); 200 μm (**F**, top); 20 μm (**E** and **F**, bottom) (**G**) Quantifications of GAD1^+^ and MAP2^+^ cells in hTH, and MAP2^+^ and NRGN^+^ cells in hFCX in control and FTLD-*GRN* cases. (**H**) Confocal and IMARIS images of PSD-95^+^, SYP^+^, and PSD-95^+^/SYP^+^ synapses near MAP2^+^ neurons in hFCX of control and FTLD-*GRN* patients. Scale bars: 7 μm (top); 4 μm (middle and bottom). (**I**) Quantification of PSD-95^+^ or SYP^+^ area in MAP2^+^ neurons in control and FTLD-*GRN* cases. (**J**) Confocal and IMARIS images of PSD-95^+^ in GFAP^+^ processes and cell bodies in L2/3 of hFCX in control and FTLD-*GRN* cases. Scale bars: 20 μm (top); 10 μm (middle and bottom). (**K**) Quantification of PSD-95^+^ area in GFAP^+^ or IBA1^+^ cells in control and FTLD-*GRN* cases. (**L**) Confocal and IMARIS images showing colocalization of PSD-95^+^ and LAMP1^+^ vesicles in GFAP^+^ astrocytes in hFCX of control and FTLD-*GRN* cases. Scale bars: 10 μm. (**M** and **N**) Confocal and IMARIS images of NF68, IBA1, and GFAP (**M**) and TDP-43 and GFAP (**N**) in hFCX of control and FTLD-*GRN* cases. Scale bars: 10 μm. All data represent mean ± SEM. Statistics in **G**, **I**, and **K** uses 2-tailed student’s *t* test, and *n* represents the number of independent samples tested. *P < 0.05, **P < 0.01, ***P < 0.005, ****P < 0.0001.

Given the robust microglial and astroglial pathology in hTH and hFCX in brains of patients with FTLD-*GRN*, we asked how this might affect neurons in the thalamocortical circuit in patients with FTLD-*GRN*. To test this, we analyzed the DEGs and their GO terms in all neuronal clusters in hTH and hFCX in brains of individuals with FTLD-*GRN* and compared them to those from mTH and mFCX in 19-month-old *Grn^–/–^* mice (Figure 6, A and B, Supplemental Figure 8, A–C, and Supplemental Tables 3 and 4). These results showed that the DEGs in most neuronal clusters in hTH and hFCX in patients with FTLD-*GRN* and mTH in *Grn^–/–^* mice were enriched with GO terms related to postsynapse, dendrite, synaptic signaling, and axon. Consistent with the modest glial pathology in mFCX in *Grn^–/–^* mice at 19 months old, only a small number of neuronal clusters in mFCX, including ExNeu-Nrgn, InNeu-Sv2c, L5, L24, and InNeu-Vip, showed significant transcriptomic changes related to these GO terms. The DEGs in hTH of the brains of individuals with FTLD-*GRN* included the upregulated genes implicated in neurodegenerative diseases, such as *APP*, *UBB*, and *DNAJB1*, and many down-regulated synaptic genes, including synaptophysin (*SYP*), synaptotagmin 1 (*SYT1*), and *GRIN2A*; whereas the DEGs in hFCX included upregulated genes, such as *MAP1A*, synapsin 1 *(SYN1*), *GRIN1*, *BIN1*, *UBB*, and *GABRA5*, and down-regulated genes, such as *BDNF*, *DCC*, *ROBO1*, *GAP43*, *NRG1*, *SYT1*, and *SYP* (Figure 6B and Supplemental Table 3).

Next, we compared the DEGs in neuronal clusters in FTLD-*GRN* with genes that were recently identified to have missplicing and inclusion of cryptic exons in brain tissues from patients with FTLD-TDP (29, 30) or in iPSC-derived neurons, where endogenous TDP-43 was depleted via CRISPR inhibition (31). These comparisons revealed many DEGs in the neuronal clusters in hTH and hFCX from patients with FTLD-*GRN*, and mTH from 19-month-old *Grn^–/–^* mice also overlapped with those genes that were misspliced or contained cryptic exons (Figure 6, C and D). In contrast, only 4 neuronal clusters in mFCX, ExNeuNrgn, InNeu-Sv2c, L5, and L2/4 showed overlapping genes (Figure 6D). Among these, *UNC13A* was downregulated in human PV^+^ and SST^+^ interneuron clusters (Figure 6B, right volcano plot), but not in clusters containing excitatory neurons. In addition, immunostains for GAD1, MAP2, and NRGN showed significant reductions in GAD1^+^, MAP2^+^, and NRGN^+^ neurons in hTH and hFCX in individuals with FTLD-*GRN* (Figure 6, E–G). Many GAD1^+^, MAP2^+^ or NRGN^+^ neurons in individuals with FTLD-*GRN* showed drastic reductions in dendritic arborization (Figure 6, E and F, bottom panels). Although the number of PV^+^ neurons was not significantly reduced in hFCX of FTLD-*GRN* cases, most PV^+^ neurons showed a drastic reduction in dendritic arbors and PV^+^ synaptic punctae (Supplemental Figure 8, D and E).

To further characterize the synaptic defects in individuals with FTLD-*GRN*, we performed immunostains using pre- and post-synaptic markers. In control hFCX, most synaptophysin^+^ (SYP^+^) and PSD-95^+^ punctae were identified in the neuropils, with very few inside neuronal cell bodies. In contrast, cortical neurons in individuals with FTLD-*GRN* contained many larger SYP^+^ and PSD-95^+^ synapses inside their cytoplasm, which were colocalized with LAMP1^+^ organelles (Figure 6, H and I and Supplemental Figure 8F). Interestingly, many GFAP^+^ astrocytes and IBA1^+^ microglia in layers 2–3 of hFCX in patients with FTLD-*GRN* also contained significantly more PSD-95^+^ punctae colocalizing with LAMP1^+^ organelles, suggesting that these glial cells were engulfing synapses (Figure 6, J–L, and Supplemental Figure 8, G and H). In addition, immunostains showed that the subcortical white matter in control hFCX contained many NEFL^+^ (NF68^+^) axons. In contrast, hFCX in individuals with FTLD-*GRN* showed a marked reduction in NEFL^+^ axons with much NEFL^+^ debris inside GFAP^+^ astrocytes (Figure 6M and Supplemental Figure 8, I–K). Finally, GFAP^+^ astrocytes in layers 2–3 of hFCX and IBA1^+^ microglia in hTH in individuals with FTLD-*GRN* were frequently near TDP-43^+^ aggregates; some even contained fragments of TDP-43 in their processes (Figures 6N and Supplemental Figure 8, L–N).

### Grn^–/–^ astrocytes promote synaptic and dendritic degeneration.

**Figure 7:**
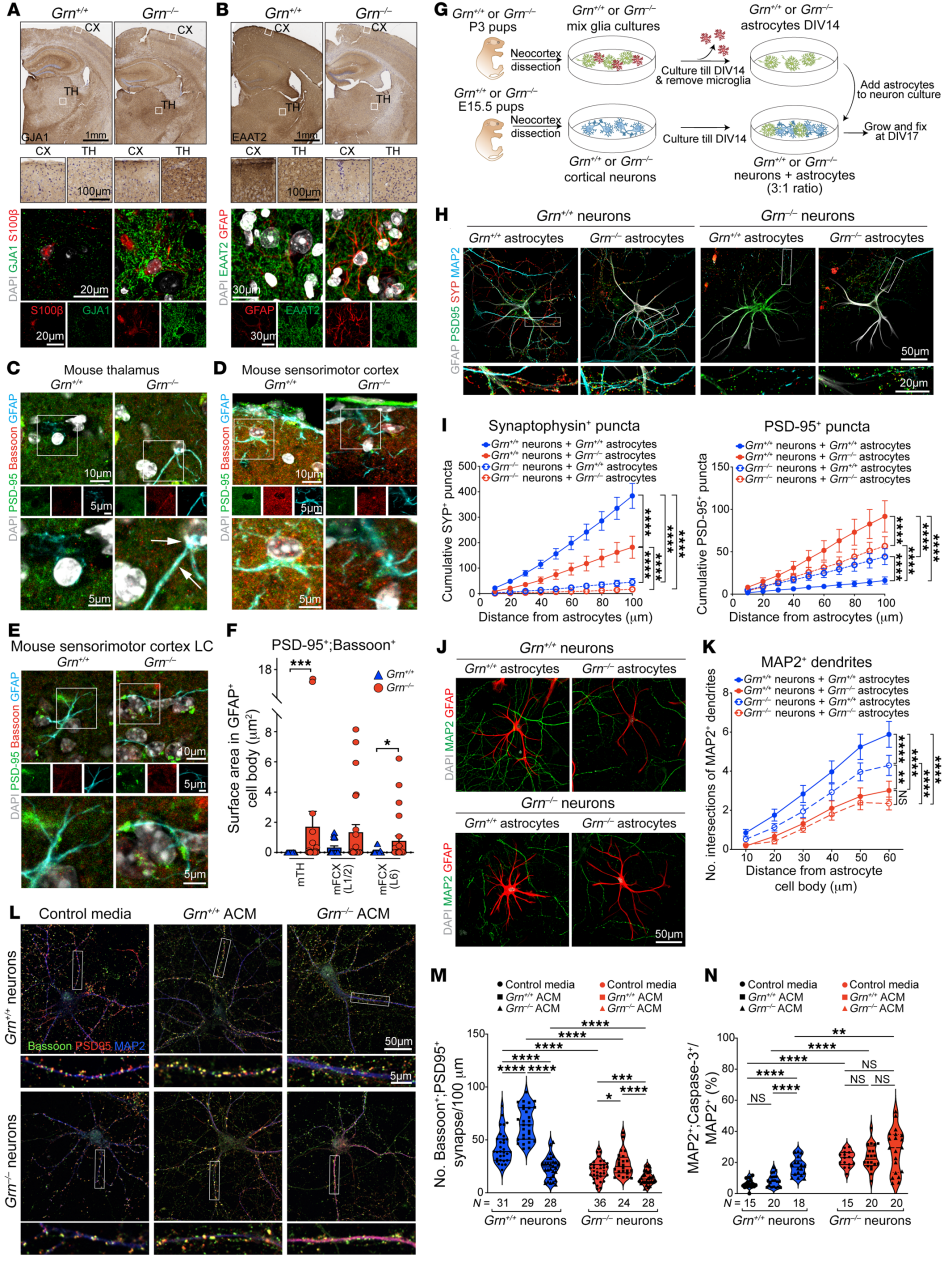
*Grn^–/–^* astrocytes promote synaptic and dendritic degeneration. (**A** and **B**). IHC and confocal images of GJA1 (A) and EAAT2 (B) in astrocytes in cortex (CX) and thalamus (TH) in 19-month-old *Grn*^+/+^ and *Grn^–/–^* mice. Scale bars: 1 mm; 100 μm (inset) (**C**–**E**) Confocal images of PSD-95, Bassoon, and GFAP in mTH (**C**) and layer 1/2 (L1/2) (**D**) and layer 6 (L6) (**E**) in mFCX in 19-month-old *Grn*^+/+^ and *Grn*^–/–^ mice. Scale bars: 10 μm; 5 μm (inset). (**F**) Quantification of PSD-95^+^;Bassoon^+^ area within GFAP^+^ astrocytes in *Grn*^+/+^ and *Grn*^–/–^ mice (*n* = 3 each). Statistics uses 2-tailed Student’s *t* test. (**G**) Schematic diagram for astrocyte-neuron cocultures. (**H**) Confocal images of MAP2, SYP, and GFAP in astrocyte-neuron cocultures. Scale bars: 50 μm and 20 μm (inset). (**I**) Quantification of cumulative SYP^+^ (left) and PSD95^+^ (right) density in *Grn*^+/+^ and *Grn*^–/–^ dendrites near *Grn*^+/+^ or *Grn*^–/–^ astrocytes. Data from 2 independent cocultures. Statistics uses 2-way ANOVA. (**J**) Confocal images of MAP2^+^ neurons and GFAP^+^ astrocytes in astrocyte-neuron cocultures. Scale bars: 50 μm. (**K**) Quantification of MAP2^+^ dendritic arborization in *Grn*^+/+^ or *Grn*^–/–^ neurons cocultured with *Grn*^+/+^ or *Grn*^–/–^ astrocytes. Data from 4 independent cocultures. Statistics uses 2-way ANOVA. (**L**) Confocal images of Bassoon, PSD-95, and MAP2 in *Grn*^+/+^ and *Grn*^–/–^ cortical neurons treated with control media, *Grn*^+/+^ ACM, or *Grn*^–/–^ ACM. Insets are higher magnification images from boxed areas above. (**M** and **N**) Quantification of Bassoon^+^;PSD-95^+^ synaptic density in the dendrites of *Grn*^+/+^ and *Grn*^–/–^ neurons (**M**) and cleaved caspase 3^+^ *Grn*^+/+^ and *Grn*^–/–^ neurons (**N**) treated with control media, *Grn*^+/+^ ACM, or *Grn*^–/–^ ACM. Data are from 4 independent cocultures. 1-way ANOVA. All quantification data represent mean ± SEM. *P < 0.05, **P < 0.01, ***P < 0.005, ****P < 0.0001. NS, not significant.

To characterize *Grn^–/–^* astrocyte phenotypes during brain aging, we performed immunostains on *Grn^+/+^* and *Grn^–/–^* mouse brain, focusing on the shared astroglial transcriptomic changes between *Grn^–/–^* mice and FTLD-*GRN* patients. These results showed significant upregulation of AQP4, APOE, and GJA1, and down-regulation of GPC5, SLC1A2 (EAAT2), SLC6A1 (GABA transporter 1 or GABATR), and GRIA2/4 in the thalamus and somatosensory cortex in *Grn^–/–^* mice at 19 months old (Figure 7, A and B and Supplemental Figure 7, H–K). Similar to the microglial phenotype, the phenotypic changes in *Grn^–/–^* astrocytes were much more prominent in the thalamus compared with the cortex. Within the cortex, *Grn^–/–^* astrocytes were more abundant in layers 1 and 2 and layers 5 and 6. Consistent with these findings, confocal images showed that many *Grn^–/–^* astrocytes in mTH and layers 1 and 2 contained PSD-95^+^;Bassoon^+^ synapses in their cell bodies (Figure 7, C–F).

To further characterize the role of PGRN-deficient astrocytes, we prepared primary cortical neuron cultures from E15.5 *Grn^+/+^* and *Grn^–/–^* mouse cortex and allowed them to mature until 14 days in vitro (DIV). In parallel, we prepared primary astrocyte cultures from P3 *Grn^+/+^* and *Grn^–/–^* mouse brains. To characterize how *Grn^–/–^* astrocytes affected the maintenance of synapses, we prepared astrocyte-neuron cocultures by adding *Grn^+/+^* or *Grn^–/–^* astrocytes to *Grn^+/+^* or *Grn^–/–^* cortical neurons at 1-to-3 ratio (Figure 7G). After 72 hours, the cocultures were harvested for a modified Sholl analysis that quantified the cumulative synaptic density surrounding the cell body of the astrocytes. When cocultured with *Grn^–/–^* astrocytes, *Grn^+/+^* and *Grn^–/–^* neurons showed drastic reductions in SYP^+^ density, but increases in PSD-95^+^ density compared with coculture with *Grn^+/+^* astrocytes (Figure 7, H–I).

Interestingly, when cocultured with *Grn^–/–^* astrocytes, both *Grn^+/+^* and *Grn^–/–^* neurons showed significant reductions in dendritic length and frequent interruptions within the radius of *Grn^–/–^* astrocytes. In contrast, the dendrites of *Grn^+/+^* and *Grn^–/–^* neurons close to *Grn^+/+^* astrocytes remained intact (Figure 7, J and K). To investigate whether *Grn^–/–^* astrocytes can also secrete factors to promote synaptic and dendritic degeneration in neurons, we collected astrocyte conditioned medium (ACM) from *Grn^+/+^* and *Grn^–/–^* astrocytes and added them to *Grn^+/+^* and *Grn^–/–^* cortical neurons. These results showed that *Grn^+/+^* ACM increased the number of Bassoon^+^;PSD-95^+^ synapses in *Grn^+/+^* and *Grn^–/–^* neurons, though this effect was more modest in *Grn^–/–^* neurons (Figure 7, L and M). In contrast, *Grn^–/–^* ACM significantly reduced the number of Bassoon^+^;PSD-95^+^ synapses in both *Grn^+/+^* and *Grn^–/–^* neurons (Figure 7, L and M). Finally, to determine the impacts of *Grn^–/–^* astrocytes on neuronal survival, we added *Grn^+/+^* and *Grn^–/–^* ACM to *Grn^+/+^* or *Grn^–/–^* neurons. As we previously reported (6), under control media, significantly more *Grn^–/–^* neurons (approximately 20%) showed cleaved caspase–3^+^ staining than *Grn^+/+^* neurons (approximately 5%). Interestingly, *Grn^–/–^* ACM modestly increased cell death in *Grn^+/+^* neurons but not in *Grn^–/–^* neurons (Figure 7N).

### Role of iPSC-derived GRN^–/–^ astrocytes in synaptic refinement, neuronal stress, and TDP-43 proteinopathy in cortical organoids.

**Figure 8:**
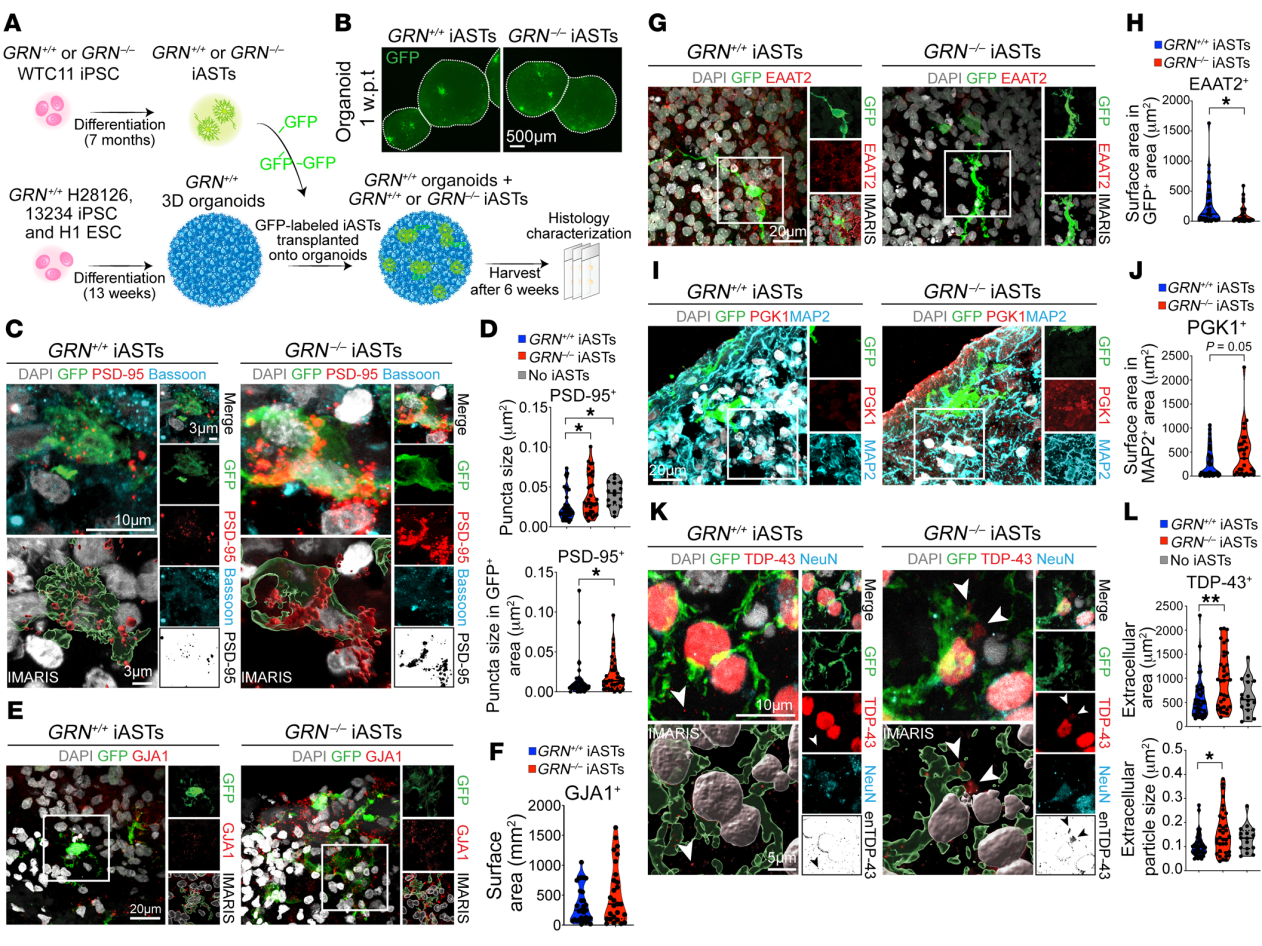
iPSC-derived PGRN-deficient astrocytes fail to promote synaptic refinement, but enhance neuronal stress and TDP-43 proteinopathy in cortical organoids. (**A**) Schematic diagram for transplanting *GRN^+/+^* and *GRN^–/–^* iPSC-derived astrocytes (iAST) to organoids. (**B**) Confocal images of GFP^+^ *GRN*^+/+^ iAST and *GRN*^–/–^ iAST in organoids 1-week after transplantation. Scale bars: 500 μm. (**C**) Confocal and IMARIS images of GFP, PSD-95, and Bassoon in *GRN*^+/+^ iAST and *GRN*^–/–^ iAST within the organoids. Scale bars: 10 μm and 3 μm (inset and IMARIS). (**D**) Quantification of PSD-95 puncta size outside and inside GFP^+^ area in organoids transplanted with *GRN*^+/+^ iAST (*n* = 10), *GRN*^–/–^ iAST (*n* = 9), or no iAST (*n* = 4). Statistics use 1-way ANOVA (upper panel) and 2-tailed Student’s *t* test (lower panel). (**E**) Confocal and IMARIS images of GFP and GJA1 in *GRN*^+/+^ iAST and *GRN*^–/–^ iAST in organoids. Scale bars: 20 μm. (**F**) Quantification of GJA1^+^ surface area in organoids with *GRN*^+/+^ iAST (*n* = 10) or *GRN*^–/–^ iAST (*n* = 9), Statistics use 2-tailed Student’s *t* test. (**G**) Confocal and IMARIS images of GFP and EAAT2 in *GRN*^+/+^ iAST and *GRN*^–/–^ iAST in organoids. Scale bars: 20 μm. (**H**) Quantification of EAAT2^+^ area in GFP^+^ *GRN*^+/+^ iAST (*n* = 10) and GFP^+^ *GRN*^–/–^ iAST (*n* = 9). Statistics use 2-tailed Student’s *t* test. (**I**) Confocal images of GFP and PGK1 in organoids transplanted with *GRN*^+/+^ or *GRN*^–/–^ iASTs. Scale bars: 20 μm. (**J**) Quantification of PGK1^+^ area in MAP2^+^ neurons in organoids transplanted with *GRN^+/+^* iASTs (*n* = 12) or *GRN*^–/–^ iAST (*n* = 10). Statistics use 2-tailed Student’s *t* test. (**K**) Confocal and IMARIS images of TDP-43 and NeuN in *GRN*^+/+^ iAST and *GRN*^–/–^ iAST-containing organoids. Scale bars: 10 μm and 5 μm (inset and IMARIS) (**L**) Quantification of TDP-43^+^ signals in extranuclear (enTDP-43) area (top) and particle size (bottom) in organoids transplanted with *GRN*^+/+^ iAST (*n* = 11), *GRN*^–/–^ iAST (*n* = 10), or no iAST (*n* = 4). 1-way ANOVA. All quantification data represent mean ± SEM, and *n* represents the number of independent samples tested. *P < 0.05, **P < 0.01.

To further characterize the role of PGRN-deficient human astrocytes in promoting neurodegeneration, we generated astrocytes from *GRN^+/+^* and *GRN^–/–^* induced–pluripotent stem cell (iPSC) lines using a previously published protocol (32). In parallel, we generated cortical organoids from *GRN^+/+^* iPSC lines (33). After *GRN^+/+^* and *GRN^–/–^* iPSC-induced astrocytes (iPSC-iASTs) became mature, they were labeled with AAV-CMV-GFP and transplanted into 13-week-old cortical organoids and cultured for another 6 weeks (Figure 8A). Using GFP and astroglial markers, including GFAP, ALDH1L1, and SOX9, we showed that both *GRN^+/+^* and *GRN^–/–^* iASTs exhibited similar grafting efficiency into the organoids (Figure 8B and Supplemental Figure 9A). Interestingly, transplanting *GRN^+/+^* and *GRN^–/–^* iASTs did not significantly alter the number of RBFOX3^+^ (NEUN^+^) or SATB2^+^ cells (callosal projection neurons), but modestly increased the number of BCL11B^+^ (CTIP2^+^) cortical neurons (Supplemental Figure 9, B–E). To examine the impact of *GRN^–/–^* iASTs, we performed immunostains using presynaptic marker Bassoon and PSD-95 and showed that neither *GRN^+/+^* nor *GRN^–/–^* iASTs affected the density of Bassoon^+^, PSD-95^+^, or Bassoon^+^;PSD-95^+^ synapses (Supplemental Figure 9, F and G). However, compared to nontransplanted organoids, the presence of *GRN^+/+^* iASTs significantly reduced the size of PSD-95^+^ synapses (Figure 8, C and D), suggesting that *GRN^+/+^* iAST promoted synaptic maturation and/or refinement. In contrast, PSD-95^+^ synapses near *GRN^–/–^* iASTs showed enlarged morphology similar to those in organoids without iAST transplantation (Figure 8, C and D).

One prominent feature of the cortical organoids is the presence of cellular stress in neurons, due to the absence of vasculature in the organoids (33, 34). To characterize the impacts of *GRN^–/–^* iASTs on cellular stress in the organoids, we used immunostaining for GJA1, glutamate transporter SLC1A2 (EAAT2), and PGK1, which is a glycolytic enzyme upregulated by the activation of the ER stress pathway. Consistent with the results from hFCX in patients with FTLD-*GRN*, *GRN^–/–^* iASTs showed a modest increase in GJA1 and reduced expression of SLC1A2 (EAAT2) (Figure 8, E–H). In addition, compared with cortical organoids transplanted with *GRN^+/+^* iASTs, organoids transplanted with *GRN^–/–^* iASTs contained more PGK1^+^ cells (Figure 8, I and J). Finally, NEUN^+^ neurons near *GRN^–/–^* iASTs showed more intense, larger, and more abundant extranuclear TDP-43 aggregates than those near *GRN^+/+^* iASTs, but no change in nuclear TDP-43 intensity (Figure 8, K and L and Supplemental Figure 9H).

## Discussion

By leveraging snRNA-Seq, histopathological validations, astrocyte-neuron cocultures, and cortical organoids, we have interrogated the effect of *GRN* mutations on glial and neuronal pathology in the thalamocortical circuit in patients with FTLD-*GRN* and *Grn^–/–^* mice. Our results support that the thalamus is a highly conserved brain region, where significant similarities in transcriptomic and histopathological changes in microglia and astrocytes promote synaptic degeneration in patients with FTLD-*GRN* and *Grn^–/–^* mice (Supplemental Figure 10). Further, we reveal a previously unappreciated role of astrocytes in promoting synaptic degeneration in the thalamus and the frontal cortex in individuals with FTLD-*GRN* and in *Grn^–/–^* mice. Finally, using astrocyte-neuron cocultures and transplantation of iPSC-derived iAST into cortical organoids, we show PGRN-deficient astrocytes promote synaptic degeneration, TDP-43 proteinopathy, and neuronal stress. Together, these results reveal parallel glial and neuronal pathology caused by PGRN deficiency in the thalamus and highlight species-specific differences in the frontal cortex.

Despite the robust microgliosis and astrogliosis in the thalamus and frontal cortex in patients with FTLD-*GRN* and 19-month-old *Grn^–/–^* mice (Figure 1), there are several distinct species- and brain region–specific differences. First, while microglia in the hTH of patients with FTLD-*GRN* share similar transcriptomic changes with those from *Grn^–/–^* mTH, microglia in the cortex of patients with FTLD-*GRN* and *Grn^–/–^* mice show no detectable overlap in their transcriptomic changes (Figure 2, H and I and Figure 4A). Second, pseudotime analysis shows that the thalamic microglia in patients with FTLD-*GRN* exhibit developmental trajectories that correlate with disease duration (Figure 4, B–D), as do thalamic and cortical astrocytes in patients with FTLD-*GRN* (Figure 5). In contrast, pseudotime analysis on cortical microglia in patients with FTLD-*GRN* does not reveal any correlation with disease duration. These results agree with those from a recent study (35), and indicate that cortical microglia in patients with FTLD-*GRN* are uniquely different. It is possible that microglia in the frontal cortex of patients with FTLD-*GRN* may have region-specific properties that accelerate them to reach end-stage disease, based on disease duration.

The discovery of the highly conserved astroglial phenotypes in the thalamus and frontal cortex in individuals with FTLD-*GRN* and in *Grn^–/–^* mice expands the repertoire of glial pathology caused by PGRN deficiency and supports that the astrocytes in patients with FTLD-*GRN* and in *Grn^–/–^* mice could contribute to neurodegeneration. For instance, these disease-associated astrocytes show significant upregulation of genes implicated in chaperone-mediated autophagy, response to toxic substances, increase in cell adhesion and glia-vascular coupling, and positive regulation of cell death (Figure 5). Furthermore, pseudotime analysis of transcriptomes of thalamic and cortical astrocytes in patients with FTLD-*GRN* reveal similar developmental trajectories that correlate with shorter disease duration, suggesting that these astrocytes are fated toward disease-specific states in multiple brain regions at much more accelerated pace compared with astrocytes in the aging mouse brain (25). Interestingly, many of these astroglial genes are also implicated in other neurodegenerative conditions, including AD (11, 17–19, 36). Finally, astrocytes in FTLD-*GRN* cases and *Grn^–/–^* mice downregulate genes critical for trans-synaptic signaling, synapse organization, and glutamate transporters (37).

In support of the transcriptomic changes, PGRN-deficient astrocytes exhibit several histopathological features suggestive of gain-of-function properties, including containing myelin debris and TDP-43 in astroglial cytoplasm; marked increases in AQP4, S100β, GJA1, and complements C3b and C4; and downregulation of glutamate transporter EAAT1 and EAAT2 (Figure 5). These features underscore the aberrant disease-specific characteristics of astrogliosis in patients with FTLD-*GRN* and *Grn^–/–^* mice during brain aging (25, 38). Indeed, astrocyte-neuron cocultures show that *Grn^–/–^* astrocytes promote synaptic and dendritic degeneration in *Grn^+/+^* and *Grn^–/–^* neurons. Furthermore, iPSC-derived *GRN^–/–^* astrocytes fail to stimulate synaptic growth and promote TDP-43 protein aggregation in cortical organoids (Figure 8). Together, these results support that PGRN-deficient astrocytes acquire disease-specific properties that contribute to neurodegeneration in patients with FTLD-*GRN* and *Grn^–/–^* mice.

One intriguing feature of FTD is that the diverse clinical entities are united by their underlying FTLD pathology (39). Indeed, patients with FTD with *GRN* mutations invariably develop abnormal TDP-43 protein aggregates, characterized by numerous TDP-43^+^ short dystrophic neurites and compact cytoplasmic inclusions in layers 2 to 3 of the frontal cortex (FTLD-TDP Type A) (39, 40). Despite this genotype-phenotype association, the exact mechanism leading to aberrant TDP-43 protein aggregate formation in patients with FTLD-*GRN* remains unclear. Results from our current study expand the role of astroglial pathology to TDP-43 proteinopathy in patients with FTLD-*GRN*. First, our results show intense astrogliosis in the thalamus and the superficial layers of the frontal cortex in patients with FTLD-*GRN*. Second, aside from the neuroanatomical correlations, many disease neurons containing cytoplasmic TDP-43 in these brain regions are surrounded by a dense meshwork of astroglial processes (Figure 6). Furthermore, TDP-43^+^ dystrophic neurites can be identified in the astroglial processes, suggesting that the reactive astrocytes in patients with FTLD-*GRN* may have engulfed these structures. Finally, cortical organoids transplanted with *GRN^–/–^* iASTs show neuronal stress and extranuclear accumulation of TDP-43 in neurons in cortical organoids (Figure 8).

The transcriptomic profiles of the neuronal clusters in the thalamus and frontal cortex of patients with FTLD-*GRN* show that the key neurodegenerative features in FTLD-*GRN* primarily affect synapses, dendrites, and axons (Figure 6). Interestingly, a recent study shows that neurons lacking nuclear TDP-43 from the frontal cortex of patients with FTLD-*C9orf72* exhibit extensive transcriptomic changes in the superficial layers (layers 1–3) of the frontal cortex (29). In addition, several DEGs in disease neurons are also targets of inclusion of cryptic exons that contain early termination codons and are subject to transcript degradation (30, 31). The most prominent DEG is *UNC13A*, which is also a disease risk gene for FTD and ALS. It is interesting to note that many GO terms defined by the DEGs in the disease neurons in FTLD-*C9orf72* overlap with those identified in the thalamus and frontal cortex from patients with FTLD-*GRN*, either via Nanostring nCounter platform or snRNA-Seq. These shared GO terms include DNA damage, transcription, and RNA splicing (Figure 1G) (29). Importantly, *UNC13A* is also identified as a significantly downregulated gene in the thalamus and frontal cortex of patients with FTLD-*GRN* (Figure 1E and Figure 5B). Given the inherent limitations of snRNA-Seq, it remains unclear whether these DEGs also have missplicing or cryptic exon inclusion. It is also unclear whether the shared DEGs in the thalamus of *Grn^–/–^* mice and individuals with FTLD-*GRN* may have species-specific splicing defects (41). Finally, our study identifies selective loss of PV^+^ inhibitory neurons in hFCX and 2 interneuron clusters in hTH in patients with FTLD-*GRN*. These results suggest that, like the thalamus in *Grn^–/–^* mice (4), the inhibitory neural circuits may also be particularly vulnerable to glial pathology in patients with FTLD-*GRN*.

## Methods

### Human iPSC lines

For cortical organoids generation, human iPSC lines, H28126 (male, Gilad Lab, University of Chicago, Chicago, Illinois, USA), 13234 (female, Conklin Lab, Gladstone Institutes) and embryonic stem cells line, H1 (male, WiCell; authenticated at source), were used. For iPSC-derived astrocytes, isogenic *GRN^+/+^* and *GRN^–/–^* human iPSC lines WTC11 (male donor, available at the Coriell Institute for Medical Research; ID no. GM25256) were provided by Bruce Conklin (Gladstone Institute of Data Science and Biotechnology, San Francisco, California, USA) and Michael E. Ward (National Institute of Neurological Disorders and Stroke, Bethesda, Maryland, USA).

### RNA integrity measurements

Snap-frozen hFCX (middle frontal gyrus at the level of mammillary body) and hTH (at the level of the subthalamic nucleus, red nucleus, and hippocampus) were sectioned on a cryostat (Leica) to collect 100 μm–thick sections for total RNA and nuclei isolation. Similarly, snap-frozen mouse brains were sectioned on a cryostat (Leica) and mFCX and mTH at the level of anterior hippocampus were micro-dissected. Total RNAs were extracted using the RNAeasy Mini kit (Qiagen) according to manufacturer’s instructions. RNA integrity was analyzed on an Bioanalyzer using RNA 6000 Nano kit (Agilent). Only samples with an RNA integrity number (RIN) of at least 6.5 were used to perform snRNA-Seq.

### NanoString nCounter gene expression analysis

Total RNA extracted from adjacent sections to the section used for snRNA-Seq were sequenced through the NanoString nCounter platform. DEGs were analyzed by nSolver software version 4.0 using the data generated by nCounter Human Neuropathology Panel and Human Neuroinflammation Panel (NanoString Technologies).

### Sample preparation for 10× snRNA-Seq

The protocol for nuclei isolation for 10× snRNA-Seq was adapted from previous studies (6, 12). Sectioned brain tissues were homogenized in 5 mL of RNAase-free lysis buffer (0.32 M sucrose, 5 mM CaCl_2_, 3 mM Mg(Ac)_2_, 0.1 mM EDTA, 10 mM Tris-HCl, 1 mM DTT, 0.1% Triton X-100 in DEPC-treated water) using glass dounce homogenizer (size A; Thomas Scientific) on ice. The homogenate was loaded into a 30 mL–thick polycarbonate ultracentrifuge tube (Beckman Coulter). Nine mL of sucrose solution (1.8 M sucrose, 3 mM Mg(Ac)_2_, 1 mM DTT, 10 mM Tris-HCl in DEPC-treated water) was added to the bottom of the tube with the homogenate. The tubes were placed in a SW28 rotor and centrifuged at 107,000*g* for 2.5 hours at 4°C. Supernatant was aspirated, and the nuclei pellets were incubated in 100 μL (for mouse samples) or 200 μL (for human samples) of DEPC-treated PBS for 20 minutes on ice before resuspending the pellet. The nuclear suspension was filtered once (for mouse samples) and twice (for human samples) through a 30 μm cell strainer (Miltenyi Biotec). Nuclei were counted before performing single-nucleus capture on the 10× Genomics Single-Cell 3′ system V2 for human samples, V3 for mouse samples. Target capture of 3,000 nuclei for human samples and 10,000 nuclei for mouse samples was used. The 10× capture and library preparation were performed using the manufacturer’s protocol. Single-nucleus libraries were sequenced on a S2 flowcell of the Illumina NovaSeq 6000 machine (with an average depth of 500 million reads/sample) at UCSF Genomics Core Facility.

### snRNA-Seq data processing, dimensionality reduction

Analyses of snRNA-Seq data were adapted from our previous studies (6, 12). Briefly, CellRanger software v.1.3.1 default parameters were used for fastq files generation, reads alignment and unique molecular identifiers (UMIs) quantification, at the exception of the use of a pre-mRNA reference file (ENSEMBL, GRCh38) to insure capturing intronic reads originating from pre-mRNA transcripts abundant in the nuclear fraction. Individual expression matrices containing numbers of UMIs per nucleus per gene were filtered to retain nuclei with at least 500 genes expressed and less than 5% of total UMIs originating from mitochondrial and ribosomal RNAs. Genes expressed in less than 3 nuclei were filtered out. In addition, mitochondrial RNAs were filtered out to exclude transcripts coming from outside of the nucleus to avoid biases introduced by nuclei isolation and ultracentrifugation. Individual matrices were combined. UMIs were normalized to the total UMIs per nucleus and log transformed. A filtered log-transformed UMI matrix was used to perform truncated singular value decomposition (SVD) with k = 50. A scree plot was generated to select the number of significant principal components (PCs) by localizing the last PC before the explained variance reaches plateau. The significant PCs were used to calculate Jaccard distance–weighted nearest-neighbor distances; the number of nearest neighbors was assigned to root square of number of nuclei. Resulting graph with Jaccard-weighted edges was used to perform Louvain clustering.

### Clustering, differential gene expression, burden score, and nuclei count

To visualize nuclei transcriptomic profiles in 2-dimensional space, *t*-distributed Stochastic Neighbor Embedding (*t*-SNE) was performed with the selected PCs and combined with cluster annotations. Cell types were annotated based on expression of known marker genes visualized on the *t*-SNE plot and by performing gene marker analysis. Primary cell type annotations of clusters were performed by comparison with previously annotated cell types, and when a repository of substantial matching was not available, a combination of literature-based annotation was used. For sub-clusters, a set of markers, specifically, overexpressed genes, was defined by differential expression analysis of the cells grouped in each subcluster against the remaining cells within the corresponding broad cell-type cluster. This analysis was applied to all cell types independently. Among the neuronal clusters, we have found that the ExNeu-NRGN cluster contained neuronal debris without definitive nuclear features, suggesting that this cluster may be a masked cell type in snRNA-Seq data sets (D. Velmeshev and A.R. Kriegstein, personal communication). Due to the very few DEGs (*n* = 2) in the “*AST-REAC*” cluster in hFCX between the control and FTLD-*GRN* samples, the gene burden score for this cluster in hFCX in FTLD-*GRN* became relatively low (5.14) compared with other clusters.

DEGs interactions were assessed using the Venn diagrams software on the Van de Peer lab website (https://www.vandepeerlab.org/software?page=2). Final Venn diagram were generated using the Eulerr.co website (https://eulerr.co/). For statistics, hypergeometric tests were performed on the Graeber Lab website (https://systems.crump.ucla.edu/hypergeometric/). GO enrichment analyses were performed using Metascape. For all cases we used the set of 17,926 protein-coding genes included in the QC data as background. All other plots including Alluvial plots, Volcano plots and Scatter plots, were generated with ggplot2 on R studio.

### DEGs in neuronal clusters and TDP-43-mediated misspliced genes

The microglial transcriptomes in the thalamus and frontal cortex in individuals with FTLD-*GRN* were compared with those from patients with AD (17–19) and with the peripheral immune cells in the CIBERSORT data sets (13). We compared the DEGs of the neuron clusters in our data to (a) the 66 alternatively spliced genes identified in NeuN^+^;TDP43^–^ compared with NeuN^+^;TDP-43^+^ nuclei reported by Ma, et al. (30); to (b) the 149 DEGs identified in NeuN^+^;TDP43^–^ compared with NeuN^+^;TDP-43^+^ nuclei reported by Liu, et al. (29); and to (c) the 192 top DEGs (filtered by *P* adjusted value = 0.001 and Log2(FC)=0.23) identified by comparison between control and TDP-43-knockdown i3Neurons reported by Brown, et al. (31).

### Pseudotime analysis

Monocle R package (Version 3) was used with default options to reconstruct trajectories of microglia and astrocytes from hFCX and hTH based on snRNA-Seq data. The initial node of each trajectory was assigned to the node enriched for control cells. Once the pseudotime was calculated, graph_test function was used to identify genes dynamically expressed along microglia and astrocyte trajectories. FDR-corrected *P* value under 0.05 was used as the cutoff to determine the dynamically expressed genes. To plot expression of genes along trajectories, normalized log transformed UMI counts for both the control and patient microglia and astrocytes was used.

### iPSC culture for cortical organoids

For cortical organoid generation, human iPSC lines, H28126 and 13234, and embryonic stem cells line, H1, were expanded on growth factor reduced (GFR) matrigel-coated 6-well plates. Stem cells were thawed in StemFlex Pro Media (Gibco) containing 10 μM Rock inhibitor Y-27632. Media was changed every other day and lines were passaged when colonies reached about 70% confluency. Stem cells were passaged using ReLeSR (Stem Cell Technologies) and residual cells manually lifted with cell lifters (Thermo Fisher Scientific). All of the cell lines used for this study were passaged between 25–40 times.

### Cortical Organoids

Cortical organoids were differentiated using the previously published directed differentiation protocol (33). Briefly, PSC lines were expanded and dissociated to single cells using Accutase Sigma A6964. After dissociation, cells were reconstituted in neural induction media at 10,000 cells per well in a 96 well v-bottom ultra-low adhesion plates. Directed differentiation protocol GMEM-based induction media included 20% Knockout Serum Replacer (KSR), nonessential amino acids, 0.11 mg/mL sodium pyruvate, penicillin-streptomycin, 0.1 mM β-mercaptoethanol, 5 μM SB431542 and 3 μM IWR1-endo. Media was supplemented with 20 μM rock inhibitor Y-27632 for the first 6 days. After 18 days, organoids were transferred from 96 well to 6 well ultra-low adhesion plates and moved onto an orbital shaker rotating at 90 rpm. Media was changed to DMEM/F12 media containing Glutamax, N2, CD Lipid Concentrate and Penicillin-Streptomycin. Throughout the culture duration, organoids were fed every other day. At 35 days, the organoids were moved into DMEM/F12-based media containing 10% FBS, 5 μg/mL heparin, N2, CD lipid concentrate and 0.5% matrigel. At 70 days, media was additionally supplemented with B27 and 1% matrigel.

### iPSC-derived astrocytes

iPSCs were cultured and maintained in Essential 8 Medium on 6 well cell culture plates coated with Vitronectin in DPBS at 5μg/ml as previously reported (32). iPSCs were dissociated and passaged using EDTA in DPBS at 0.5mM. Human iPSCs (WTC11) were grown on vitronectin-coated tissue culture plates using Essential 8 media. On day 0 of differentiation, iPSCs were dissociated into small aggregates averaging 50μm in diameter and transferred into untreated tissue culture flasks with Neurosphere Induction Media (NSIM) (DMEM-F12/Neurobasal-A at 1:1, N2 Supplement, B27-Vit.A Supplement, MEM-NEAA) plus SMAD inhibitors SB431542 and DMH1. On day 7, once embryoid bodies began to show rosette clusters indicating early neuroepithelia morphological hallmarks, spheroids were transferred to matrigel coated tissue culture plates with NSIM and SMAD inhibitors were removed. Media was changed every 24 hours until the outer-most migratory cells had attached to the surrounding area of each spheroid, exposing the rosette clusters within. On day 14, rosette clusters were mechanically removed and transferred to untreated tissue culture flasks with NSIM + FGFb at 10 ng/mL. On day 20, spheroids were triturated into a single cell suspension and transferred to a new untreated cell culture flask with astrocyte media (ASM) (DMEM-F12, N2 Supplement, B27-Vit.A Supplement, Heparin plus Y27632) at 10μM. From Day 28 to 180, spheroid aggregates were maintained in suspension with ASM + EGF and FGFb with media changes every 4–5 days. Spheroid aggregates were triturated every 7–10 days and transferred to new tissue culture flasks.

### iAST transplantation

Before transplantation, iASTs were dissociated and resuspended at 1,000,000 cells/mL. Cells were pelleted and resuspended in organoid medium containing AAV-CMV-GFP at a concentration of 1:1,000 to 1:2,000 to label dissociated cells for 45 minutes to 1 hour at 37°C. Cells were washed 3 times for 5 minutes and spun down at 300*g* for 5 minutess. iAST were resuspended in the cortical organoid media without FBS and with 0.1% rock inhibitor. Cortical organoids, which had been cultured for 13 weeks, were placed into 6-well plates containing a Millicell inserts (Millipore). Half of the media was removed to stabilize the organoids on the insert. Each organoid received 125,000 iAST, and, following the transplantation, the organoids were incubated for 2 hours to allow the iAST to incorporate into the organoids. Organoids were left undisturbed for 4 days, then transferred to low adhesion 6-well plates and collected for immunohistochemistry 6 weeks after transplantation.

### Primary astrocyte cultures and astrocyte conditioned media

Cerebral cortex from P3–4 *Grn^+/+^* and *Grn^–/–^* mice were dissected and cultured in DMEM media with 20% FBS and 20 ng/mL GM-CSF. After 10–12 days in culture (DIV10–12), microglia were removed from the mixed-glia cultures by shaking at 200 rpm for 2 hours and primary astrocytes were detached with trypsin and cultured in the serum-free conditioned medium (50% Neurobasal media and 50% DMEM with 1mM sodium pyruvate, 2 mM L-glutamate, 5 μg/mL N-acetyl-L-cysteine, 100 μg/mL BSA, 100 μg/mL transferrin, 16 μg/mL putrescine, 60ng/mL progesterone, 40 ng/mL sodium selenite and 5 ng/mL HB-EGF) for 72 hours. The conditioned media from astrocytes was concentrated with Ultracel 10K (Amicon) and the final protein concentrations measured using BCA assay kit (Thermo Fisher Scientific). Primary cortical neurons were prepared from the cerebral cortex of E15.5 *Grn*^+/+^ and *Grn*^–/–^ embryos as previously described (4, 6).

### Immunohistochemistry staining, imaging, and counting

Immunohistochemical stains were performed on 40 μm free-floating sections of 4% PFA-fixed mouse brains and 16 μm mounted sections of 4% PFA-fixed human brains prepared using a Leica cryostat. The staining protocol included antigen retrieval treatment by incubating tissue sections in 10 mM sodium citrate (pH 6.0) at 90°C for 10 minutes. Immunostains were developed using DAB and counterstained with Nissl or hematoxylin. Information on primary antibodies is provided in Supplemental Table 5. Images were captured using an Aperio ImageScope (Leica Biosystems) with a 20× objective zoom ×2. GAD1^+^, PV^+^, NGRN^+^, and MAP2^+^ neurons in human tissue were counted using Neurolucida 2017 (MBF Biosciences) on a PC attached to an Olympus BX51 microscope with a 20× objective. IBA1^+^ microglia and GFAP^+^ astrocytes in human and mouse tissues were counted using an optical fractionator–based method using Stereology Investigator 2017 (MBF Biosciences) on a PC attached to an Olympus BX51 microscope with a 60× objective and a motorized XYZ stage.

### Immunofluorescence staining, image acquisition, and 3D-reconstruction

All human, mouse, and organoid samples were fixed, submerged in sucrose solution, and embedded in OCT. Immunofluorescence staining was performed on 40 μm free-floating sections of 4% PFA-fixed mouse brains and 16 μm mounted sections of 4% PFA-fixed human brains, and 12 μm mounted sections of 4% PFA-fixed organoids were prepared using a Leica cryostat. The staining protocol included antigen retrieval treatment by incubating tissue sections in 10mM sodium citrate (pH 6.0) at 90°C for 10 minutes. DAPI was used for fluorescent nuclear counterstaining. Lipofuscin autofluorescence was reduced by a 10-minute treatment of Sudan Black 1% in 70% ethanol. Z-stacks were taken on a Nikon C2 confocal microscope using the NIS-Elements AR 5.30.02 software at 60× with a step of 1 μm. 3D reconstruction was done using IMARIS ×64 (Oxford Instruments). Images were analyzed in ImageJ.

### Data availability

The FASTQ files generated by snRNA-Seq from individuals with and without FTLD-*GRN* and from the sensorimotor cortex of 19-month-old *Grn^+/+^* and *Grn^–/–^* mice have been deposited at the NIH sequence read archive (SRA; accession no. PRJNA644744 [human] and PRJNA922058 [mouse]).

### Statistics

For transcriptomics, the exact numbers of human and mouse samples are indicated in the figure legends. Four mice per brain region and genotype were used to perform snRNA-Seq. Nine to 11 samples per brain region were used as control or disease cases for snRNA-Seq after RIN quality filter. DEG statistics were performed using Moran’s *I* tests. Gene burden score statistics were calculated using 1-way ANOVA test for parametric data and Kruskal-Wallis test for non-parametric data. Venn diagram statistics comparison were assessed using hypergeometric tests. *P* values for genes dynamically expressed along the pseudotime trajectories were obtained thanks to the ‘graph_test’ function. GO-terms *P* values were from the Metascape analysis. The number of nuclei in each cluster was normalized to the total number of nuclei captured from each individual. For each cluster, nuclei count normality was assessed for individuals with and without FTLD-*GRN* and, if normally distributed, 2-tailed unpaired Student’s *t* tests were performed, if nonnormally distributed, Mann-Whitney *U* tests were performed.

For immunostaining quantification, the exact numbers of samples and images used are indicated in the figure legends. Statistical analyses were done using Prism 9.0 (GraphPad). For comparisons between 2 groups, if normally distributed, 2-tailed unpaired Student’s *t* tests were performed. If nonnormally distributed, Mann-Whitney *U* tests were performed. For multiple comparisons with 1 factor, if normally distributed, 1-way ANOVAs with multiple comparisons were performed. If nonnormally distributed, Kruskal-Wallis tests with multiple comparisons were performed. For multiple comparisons with 2 factors, 2-way ANOVAs with multiple comparisons were performed. Posthoc analyses were performed using Tukey’s multiple comparison tests.

### Study approval

#### Human brain tissue collection.

Deidentified postmortem brain tissues from the hTH and hFCX of patients with FTLD-*GRN* were obtained from the Neurodegenerative Disease Brain Bank at the UCSF. Age-matched controls from the same brain regions were obtained from the Autopsy Service in the Department of Pathology at the UCSF and from the Mount Sinai Brain Bank via NIH NeuroBioBank. Brain tissue samples were collected after informed consent was obtained from the patient or their families, in accordance with guidelines put forth in the Declaration of Helsinki. Autopsy consent and all protocols were approved by the Human Gamete, Embryo, and Stem Cell Research Committee and the IRB at UCSF. All control cases received extensive neuropathological evaluations to rule out the presence of neurological diseases. Detailed information regarding age, gender, postmortem interval, and mutations in the *GRN* gene for FTLD-*GRN* cases is provided in Supplemental Table 1.

#### Mouse brain tissue collection.

All mice experiments were conducted in accordance with the UCSF IACUC (Protocol no. AN169548). Mice carrying deletion of the exons 2–13 of the mouse progranulin gene (*Grn*^–/–^) were previously reported by our laboratory (4, 6, 42). Both male and female mice were used.

## Author contributions

EM and EJH conceived the project and designed the experiments. EM performed and analyzed the Nanostring nCounter experiments. EM performed snRNA-Seq on human samples and supervised JB on snRNA-Seq on 19-month-old *Grn^+/+^* and *Grn^–/–^* mouse samples. EM, DV, and RKP performed bioinformatics analyses on the snRNA-Seq data. EM, AR, CM, and JZ performed immunohistochemistry, image analyses and the quantification of glial and neuronal pathology, astrocyte-neuron cocultures, and organoid transplants. EM and MGA performed organoid experiments, MK, MdM and EMU provided iPSC-derived astrocytes and related reagents. AR analyzed the results from the organoids with supervision from EM. ANL, SS, LTG, WWS, and BLM contributed to the collection of postmortem brain tissues from individuals with FTLD-*GRN*. MFK and ARK contributed reagents and expertise. EM and EJH wrote the manuscript with input from all authors.

## Supplementary Material

Supplemental data

Supplemental tables 1 and 5

Supplemental table 2

Supplemental table 3

Supplemental table 4

## Version 1 01/05/2023

In-Press Preview

## Version 2 03/15/2023

Electronic publication
