# Supplementary material for: Astroglial toxicity promotes synaptic degeneration in the thalamocortical circuit in frontotemporal dementia with GRN mutations
Source: J Clin Invest. 2023 Mar 15;133(6):e164919. doi: 10.1172/JCI164919 (PMC10014110; doi:10.1172/JCI164919)
Supplement: Supplemental data [file jci-133-164919-s061.pdf]

Astroglial toxicity promotes synaptic degeneration in the thalamocortical circuit in frontotemporal dementia with *GRN* mutations

Elise Marsan, Dmitry Velmeshev, Arren Ramsey, Ravi K. Patel, Jiasheng Zhang, Mark Koontz, Madeline G. Andrews, Martina de Majo, Cristina Mora, Jessica Blumenfeld, Alissa N. Li, Salvatore Spina, Lea T. Grinberg, William W. Seeley, Bruce L. Miller, Erik M. Ullian, Matthew F. Krummel, Arnold R. Kriegstein, Eric J. Huang

SUPPLEMENTARY FIGURES 1-10 AND LEGENDS

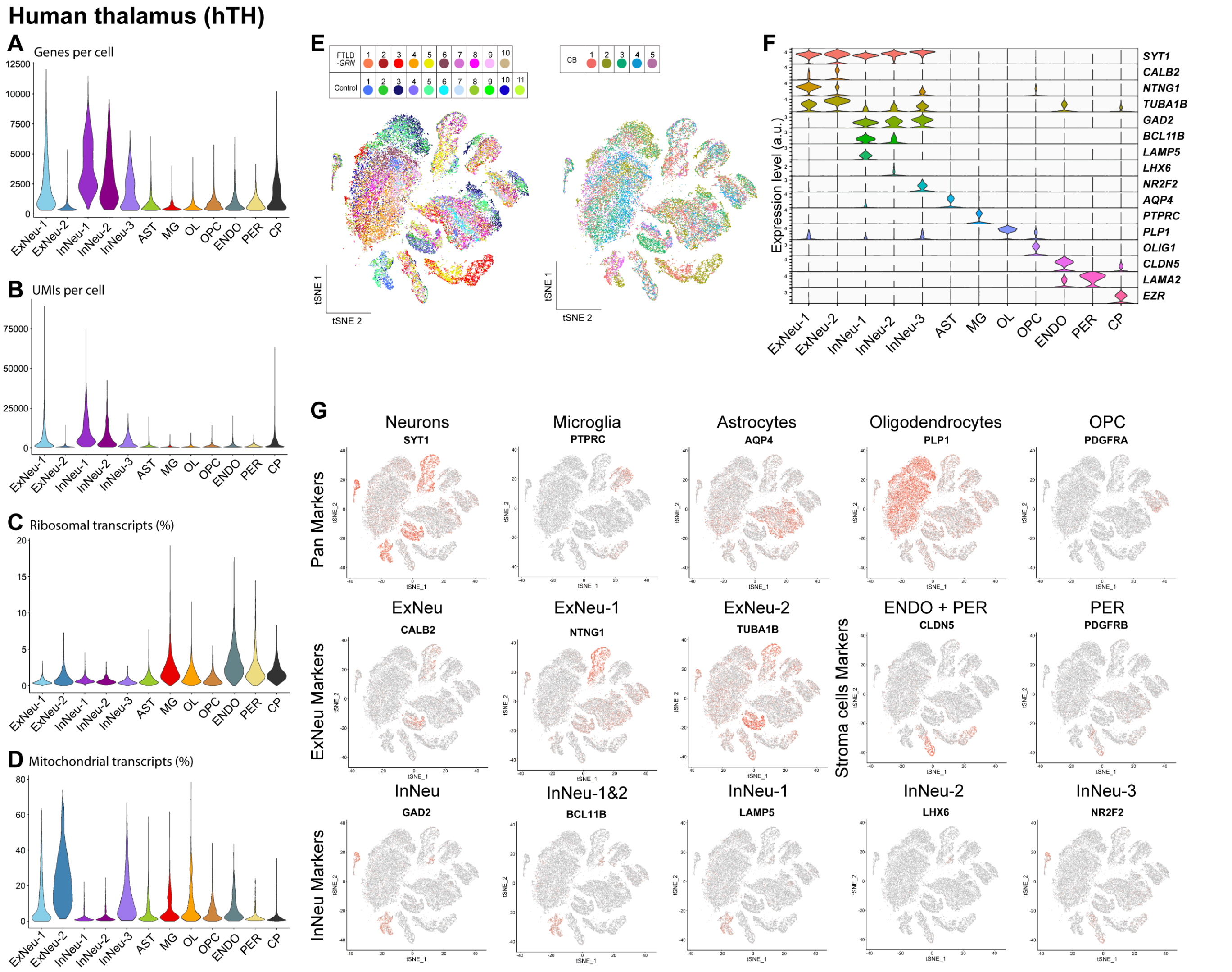

Supplementary Figure 1. Cell type identification in human thalamus. A. Genes count per cell. **B.** Unique molecular identifiers (UMIs) count per cell. **C.** Percentage of ribosomal transcripts per cell. **D.** Percentage of mitochondrial transcripts per cell. **E.** *t*SNE plots representing each human case's thalamic cells. **F–G.** Violin plots (F) and *t*SNE plots (G) showing cell subtype-specific markers for each cluster.

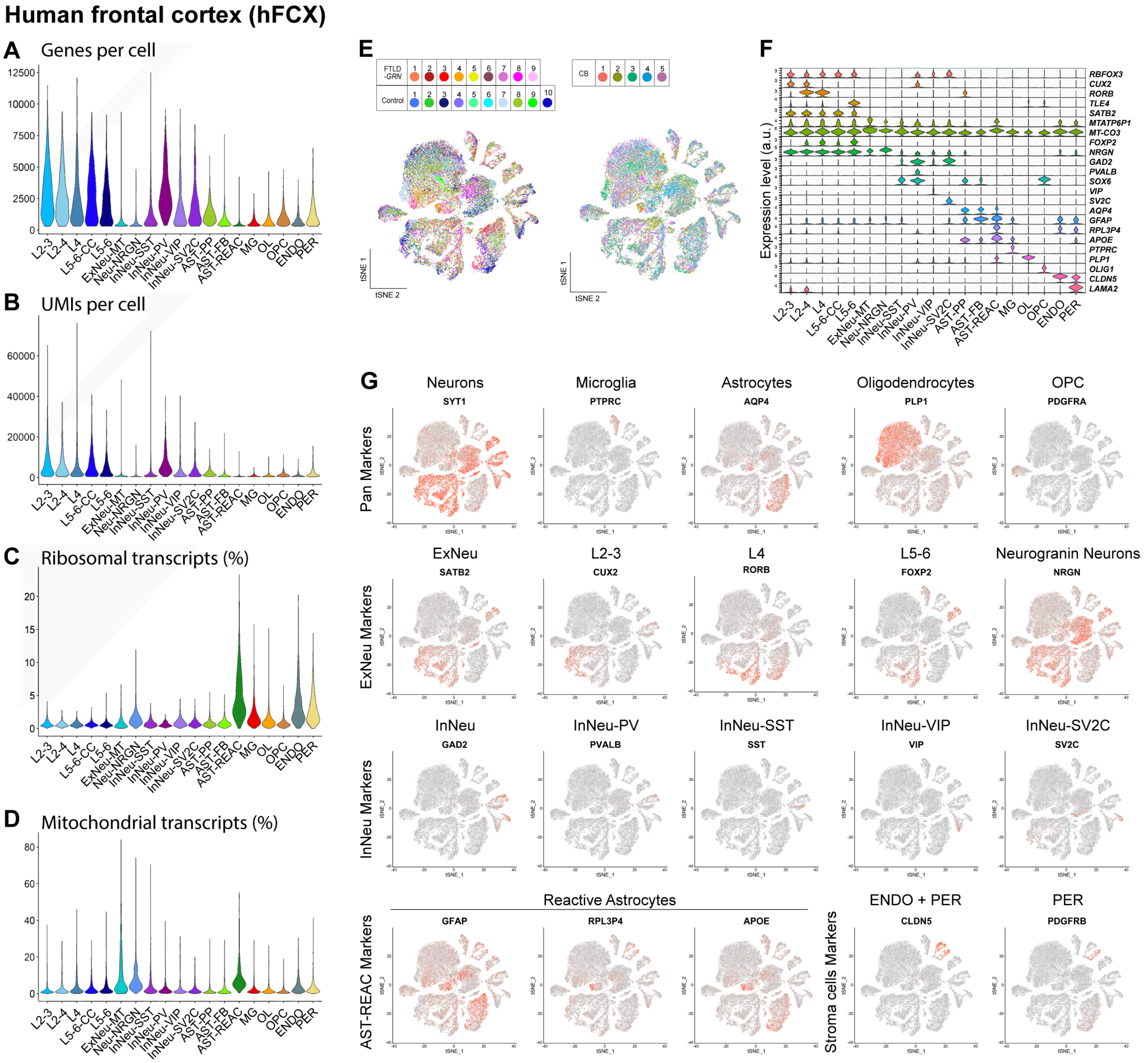

Supplementary Figure 2. Cell type identification in human frontal cortex. A. Genes count per cell. **B.** Unique molecular identifiers (UMIs) count per cell. **C.** Percentage of ribosomal transcripts per cell. **D.** Percentage of mitochondrial transcripts per cell. **E.** *t*SNE plots representing each human case's cortical cells. **F–G.** Violin plots (F) and *t*SNE plots (G) showing cell subtype-specific markers for each cluster.

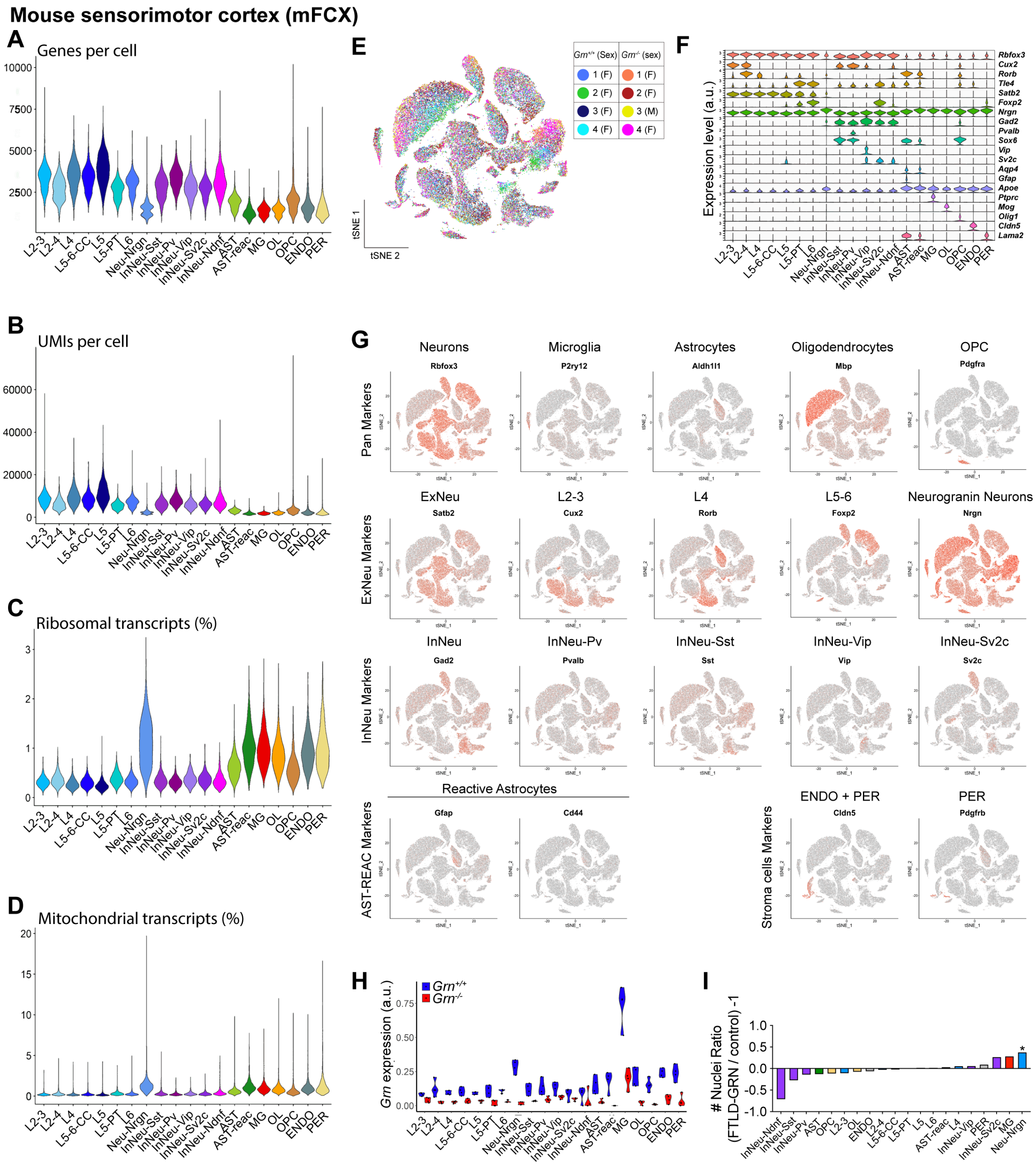

Supplementary Figure 3. Cell type identification in mouse frontal cortex. A. Genes count per cell. **B.** Unique molecular identifiers (UMIs) count per cell. **C.** Percentage of ribosomal transcripts per cell. **D.** Percentage of mitochondrial transcripts per cell. **E.** *t*SNE plots representing each mouse cortical cells. **F–G.** Violin plots (F) and *t*SNE plots (G) showing cell subtype-specific markers for each cluster. **H.** Violin plots showing *Grn* mRNA expression in the mouse frontal cortex (mFCX) cell types. **I.** Bar graphs showing the ratio of the number of nuclei captured in the mFCX in *Grn*$^{+/+}$ and *Grn*$^{-/-}$ mice. * P < 0.05, parametric (OL, L6, L2-3, L2-4, L4, Neu-Nrgn, AST, InNeu-Sv2c, InNeu-Vip, L5-6-cc, ENDO, L5, MG, InNeu-Pv, OPC, InNeu-Sst) or non-parametric Student's *t* test (PER, AST-reac, L5-PT, InNeu-Ndnf). For non-significant comparisons, no symbol is assigned.

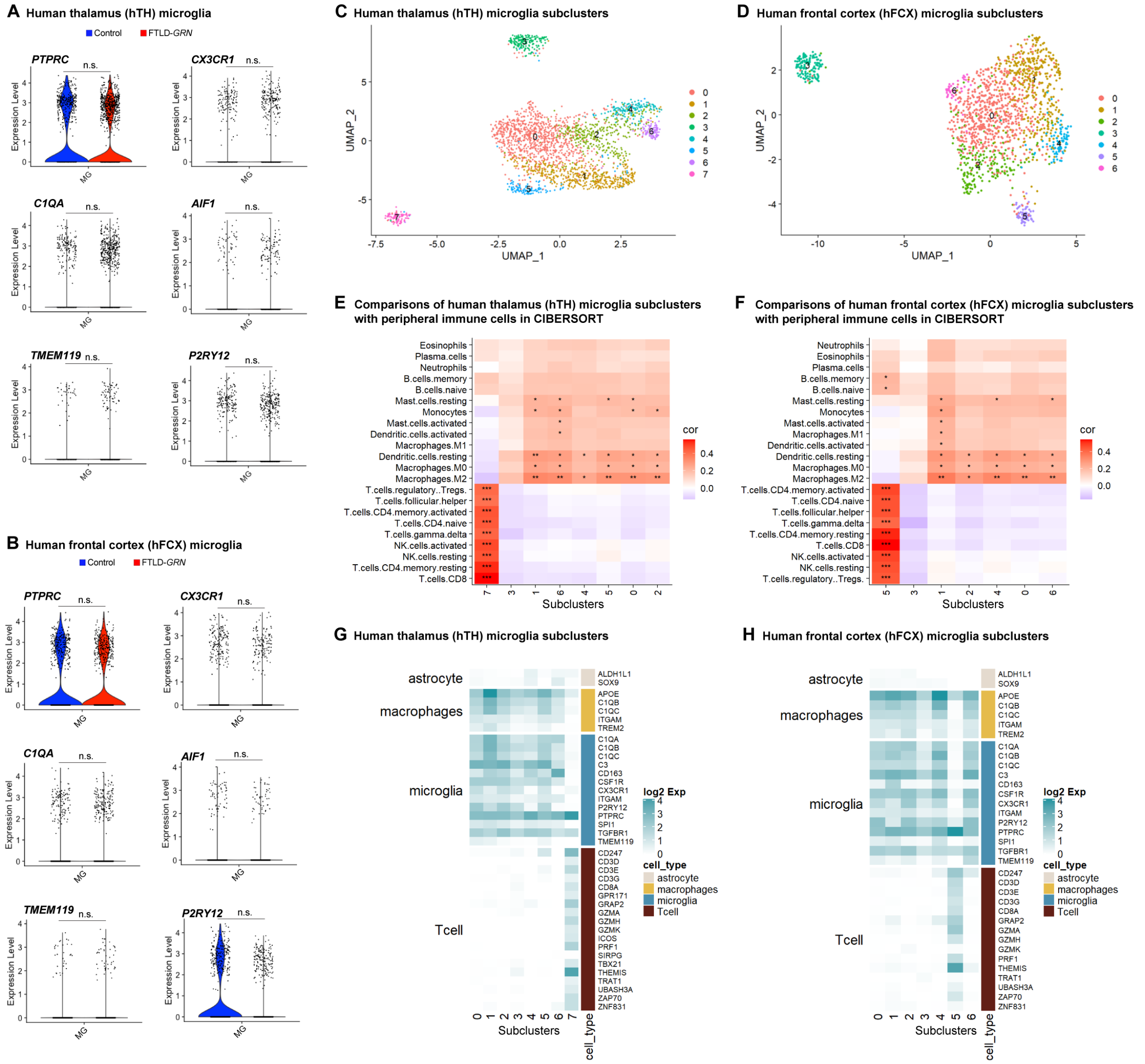

Supplementary Figure 4. Characterizations of hTH and hFCX microglia and comparison with peripheral immune cells. A-B. Violin plots for homeostatic microglial gene in human thalamus (hTH) (A) and human frontal cortex (hFCX) (B) in control and FTLD-*GRN* patients. **C-D.** UMAPs showing the microglia sub-clusters in hTH (C) and in hFCX (D). **E-F.** Heatmaps showing the comparisons between the genes expressed in the hTH microglia sub-clusters (E) or hFCX microglia sub-clusters (F) with gene expression signatures in peripheral immune cells available in CIBERSORT. The scale indicates the correlation coefficient scores of gene expression. **G-H.** Heatmaps showing the expression level of homeostatic astrocytes, microglia, macrophages, and T-cell genes in the different microglia subclusters from hTH (F) and hFCX (H). The scale indicates the relative expression level (log2). All quantification data represent mean ± SEM. ns, not significant.

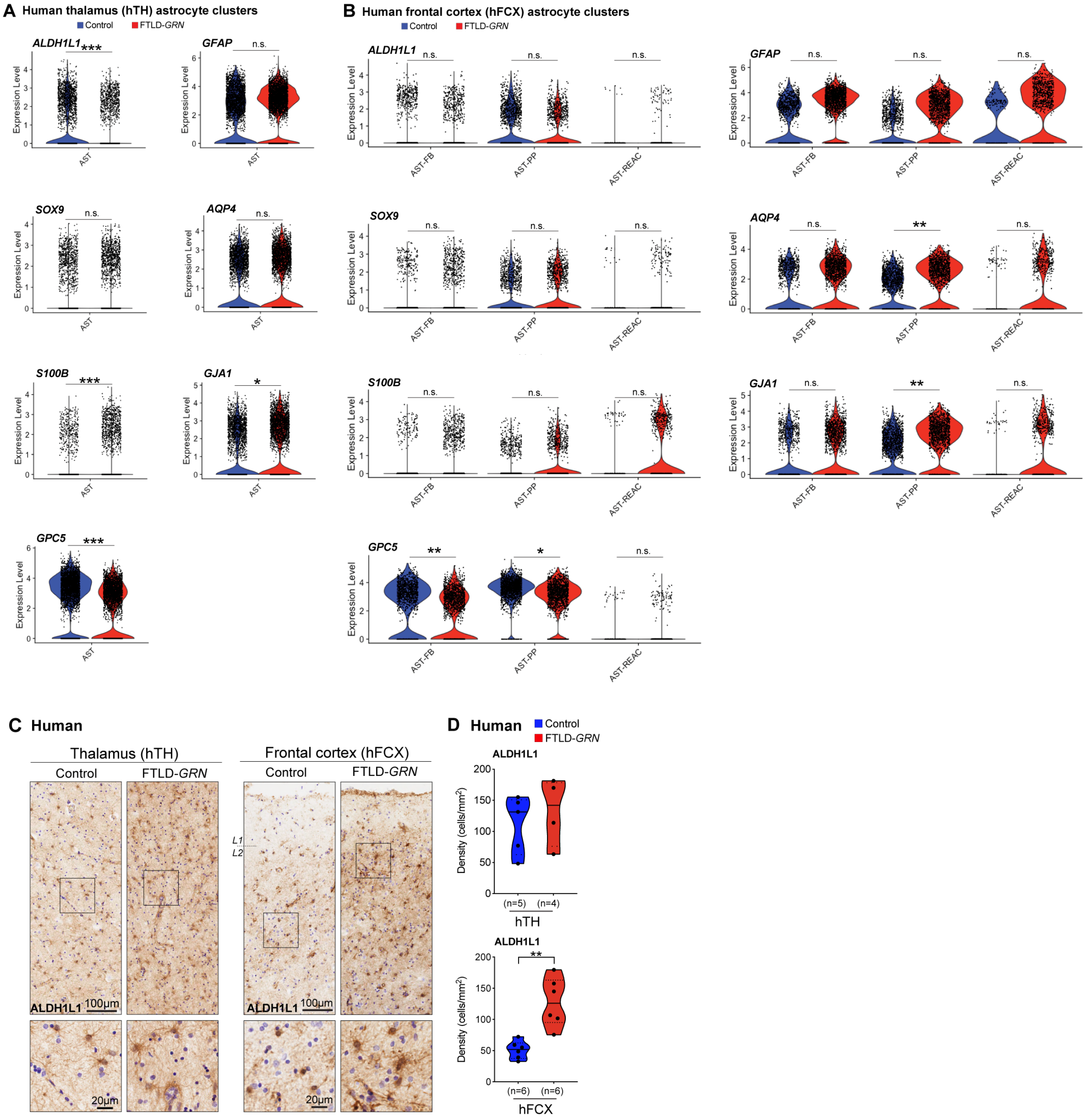

Supplementary Figure 5. Characterizations of hTH and hFCX astrocytes. A-B. Violin plots for homeostatic astroglial genes in human thalamus (hTH) (A) and human frontal cortex (hFCX) (B) in control and FTLD-*GRN* cases. **C.** Immunohistochemical stains for ALDH1L1 in hTH and hFCX in control and FTLD-*GRN* patients**. D.** Quantification of ALDH1L1^{+} astrocyte density in human thalamus (hTH) and human frontal cortex (hFCX). Statistics use Student's *t* test. All quantification data represent mean ± SEM. * P < 0.05, ** P < 0.01, ns, not significant.

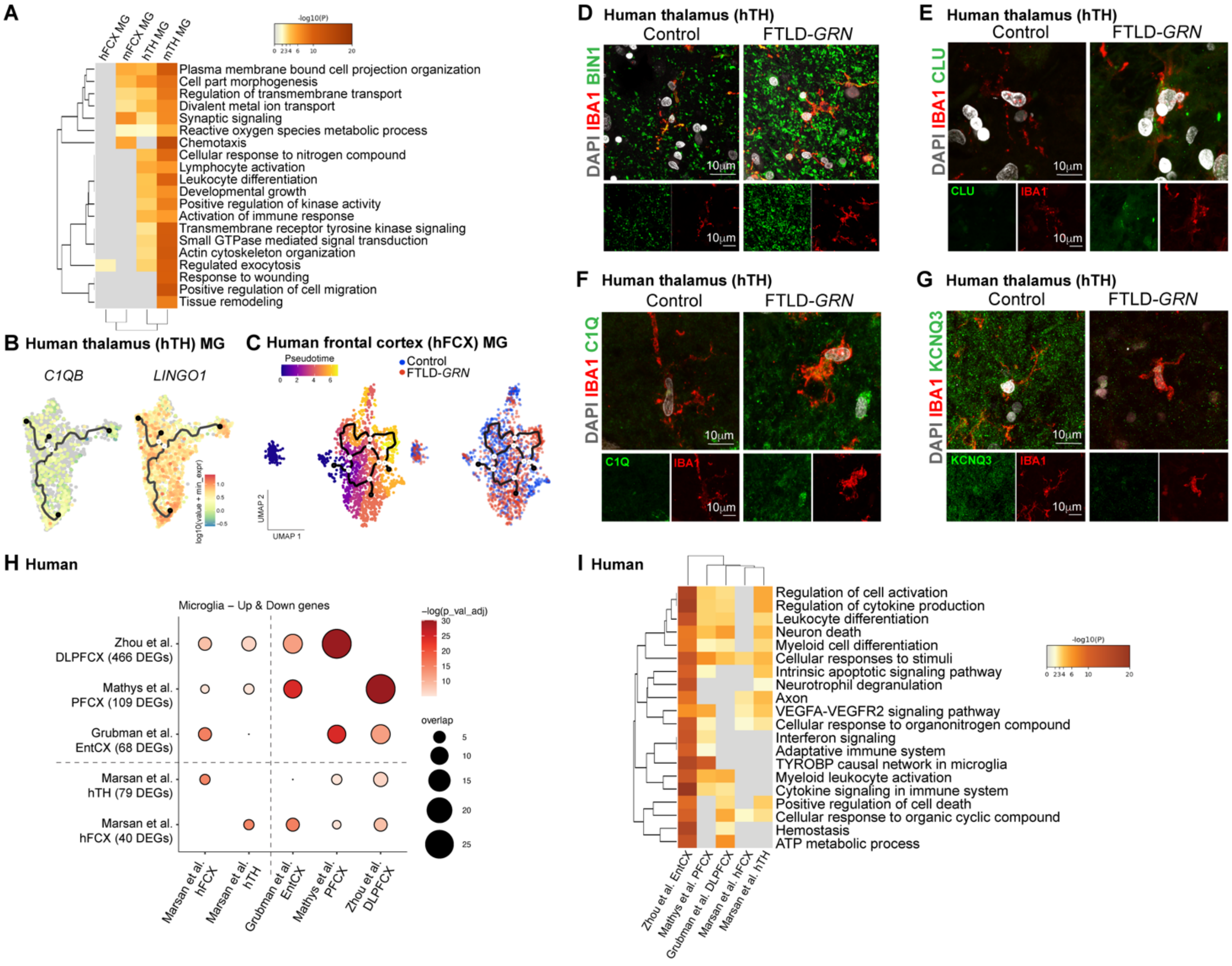

Supplementary Figure 6. Characterization of the transcriptomic and phenotypic changes in PGRN-deficient microglia. A. Heatmap comparing the top 20 gene-ontology (GO) terms defined by DEGs in the MG clusters in human and mouse thalamus and frontal cortex. The color scale indicates -Log10 P-value of the GO terms calculated based on the accumulative hypergeometric distribution. **B.** Relative expression levels of *C1QB* and *LINGO1* transcripts projected onto the UMAP space for the MG cluster in hTH. Scale represents Log10 of the expression of representative genes. **C.** Pseudotime analysis of the MG clusters in hFCX projected on UMAP (left panel) or based on diagnosis (control vs. FTLD-*GRN* patients)(right panel). **D-G.** Confocal images for BIN1, CLU, C1Q, KCNQ3, and IBA1 in IBA1+ microglia in the frontal cortex and thalamus of control and FTLD-*GRN* patients. **H.** Comparison of differentially expressed genes (DEGs) in microglial clusters from FTLD-*GRN* patients to those from the microglial clusters in the prefrontal and entorhinal cortex from Alzheimer's disease patients. **I.** Heatmap showing the overlap between the GO terms defined by DEGs in panel H.

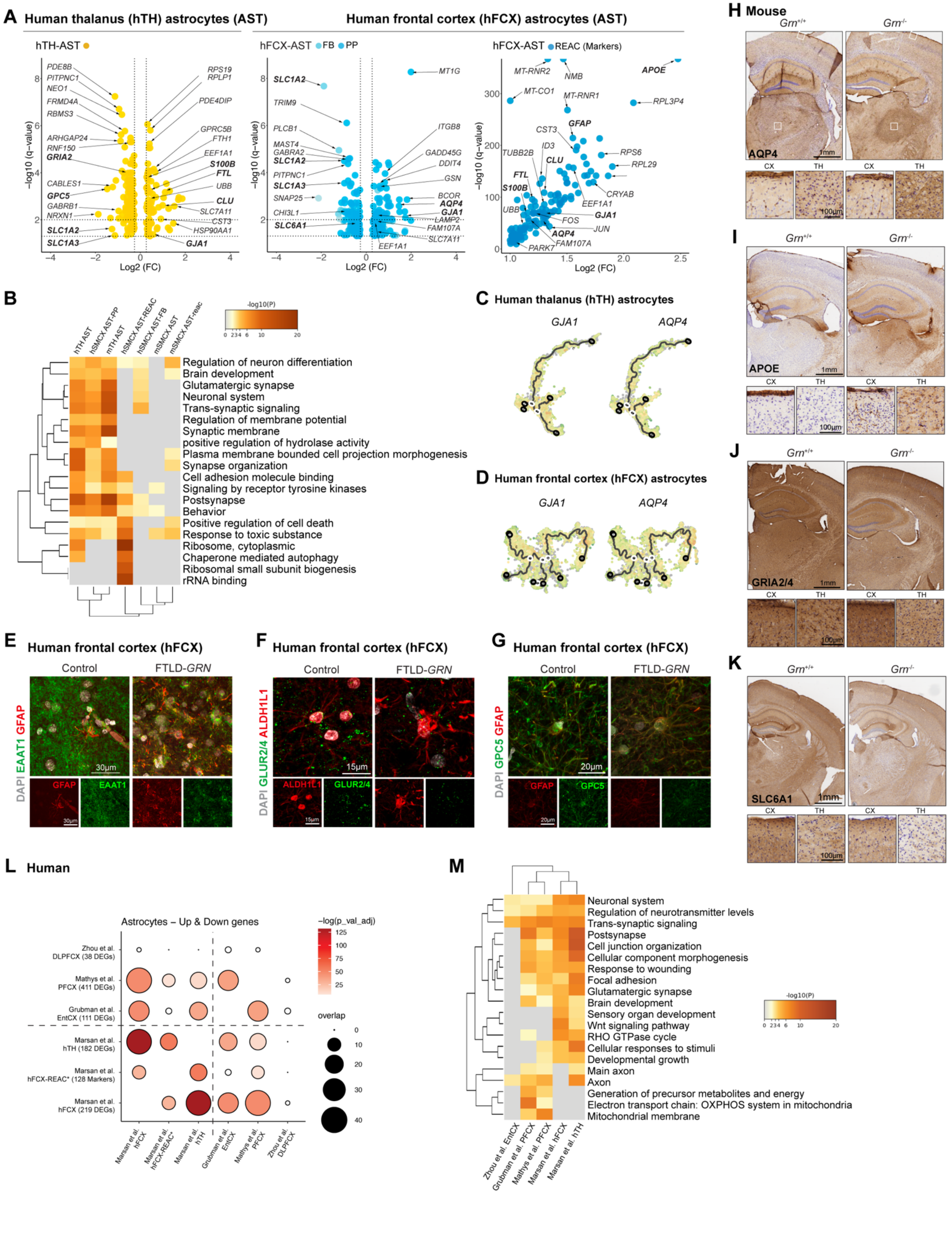
A
Human thalanus (hTH) astrocytes (AST)
Human frontal cortex (hFCX) astrocytes (AST)
hTH-AST
hFCX-AST FB PP
hFCX-AST REAC (Markers)
−log10 (q−value)
Log2 (FC)
PDE8B
PITPNC1
NEO1
FRMD4A
RBMS3
ARHGAP24
RNF150
GRIA2
CABLES1
GPC5
GABRB1
NRXN1
SLC1A2
SLC1A3
RPS19
RPLP1
PDE4DIP
GPRC5B
FTH1
EEF1A1
S100B
FTL
UBB
CLU
SLC7A11
CST3
HSP90AA1
GJA1
TRIM9
PLCB1
MAST4
GABRA2
PITPNC1
SNAP25
CHI3L1
SLC6A1
MT1G
ITGB8
GADD45G
DDIT4
GSN
BCOR
AQP4
LAMP2
FAM107A
MT-RNR2
NMB
APOE
MT-CO1
MT-RNR1
RPL3P4
CST3
GFAP
TUBB2B
ID3
RPS6
RPL29
CRYAB
FOS
JUN
PARK7
B
-log10(P)
hTH-AST
hSMCX AST-PP
mTH AST
hSMCX AST-REAC
hSMCX AST-FB
mSMCX AST
mSMCX AST-reac
Regulation of neuron differentiation
Brain development
Glutamatergic synapse
Neuronal system
Trans-synaptic signaling
Regulation of membrane potential
Synaptic membrane
positive regulation of hydrolase activity
Plasma membrane bounded cell projection morphogenesis
Synapse organization
Cell adhesion molecule binding
Signaling by receptor tyrosine kinases
Postsynapse
Behavior
Positive regulation of cell death
Response to toxic substance
Ribosome, cytoplasmic
Chaperone mediated autophagy
Ribosomal small subunit biogenesis
rRNA binding
C Human thalanus (hTH) astrocytes
GJA1
AQP4
D Human frontal cortex (hFCX) astrocytes
GJA1
AQP4
E Human frontal cortex (hFCX)
Control
FTLD-GRN
DAPI EAAT1 GFAP
30μm
F Human frontal cortex (hFCX)
DAPI GLUR2/4 ALDH1L1
15μm
G Human frontal cortex (hFCX)
DAPI GPC5 GFAP
20μm
H Mouse
Grn+/+
Grn-/-
AQP4
1mm
CX
TH
100μm
I
APOE
J
GRIA2/4
K
SLC6A1
L Human
Astrocytes – Up & Down genes
−log(p_val_adj)
overlap
Zhou et al. DLPFCX (38 DEGs)
Mathys et al. PFCX (411 DEGs)
Grubman et al. EntCX (111 DEGs)
Marsan et al. hTH (182 DEGs)
Marsan et al. hFCX-REAC* (128 Markers)
Marsan et al. hFCX (219 DEGs)
M
Neuronal system
Regulation of neurotransmitter levels
Trans-synaptic signaling
Postsynapse
Cell junction organization
Cellular component morphogenesis
Response to wounding
Focal adhesion
Glutamatergic synapse
Brain development
Sensory organ development
Wnt signaling pathway
RHO GTPase cycle
Cellular responses to stimuli
Developmental growth
Main axon
Axon
Generation of precursor metabolites and energy
Electron transport chain: OXPHOS system in mitochondria
Mitochondrial membrane
Zhou et al. EntCX
Grubman et al. PFCX
Mathys et al. PFCX
Marsan et al. hFCX
Marsan et al. hTH

Supplementary Figure 7. Characterization of the transcriptomic and phenotypic changes in PGRN-deficient astrocytes. A. Volcano plots showing differentially expressed genes (DEGs) in hTH astrocytes (left panel) and in hFCX astrocytes FB and PP (middle), and gene markers in hFCX reactive astrocyte cluster (right). **B.** Heatmap comparing the top 20 gene-ontology (GO) terms defined by DEGs in the AST clusters in human and mouse thalamus and frontal cortex. The color scale indicates -Log10 P-value of the GO terms calculated by the accumulative hypergeometric distribution. **C.** Relative expression levels of *GJA1* and *AQP4* transcripts projected onto the UMAP space for the AST cluster in hTH. Scale represents Log10 of the expression of representative genes. **D.** Relative expression levels of *GJA1* and *AQP4* transcripts projected onto the UMAP space for the AST clusters in hFCX. Scale represents Log10 of the expression of representative genes. **E-G.** Confocal images for EAAT1, GLUR2/4, and GPC5 in GFAP$^+$ astrocytes in hFCX in control and FTLD-*GRN* patients. **H-K.** Immunohistochemical stains for AQP4 (H), APOE (I), GRIA2/4 (J), and SLC6A1 (K) in 19-month-old *Grn*$^{+/+}$ and *Grn*$^{-/-}$ mouse brain. CX, cerebral cortex; TH, thalamus. **L.** Comparison of DEGs in astrocyte clusters from FTLD-*GRN* patients to those from the astrocyte clusters in the prefrontal and entorhinal cortex from Alzheimer's disease patients. **M.** Heatmap showing the overlap between the GO terms defined by DEGs in panel L.

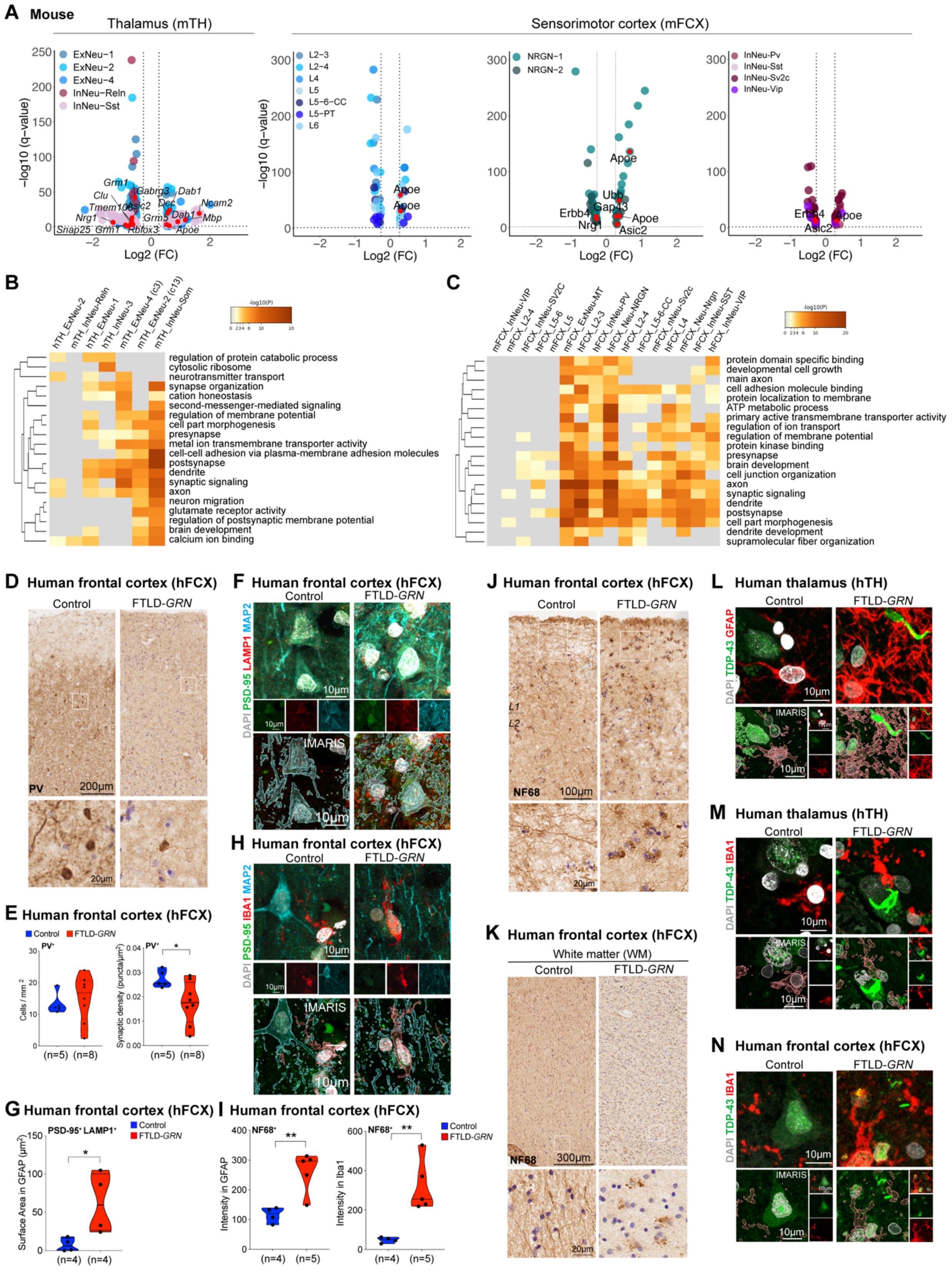
A Mouse
Thalamus (mTH)
Sensorimotor cortex (mFCX)
ExNeu-1
ExNeu-2
ExNeu-4
InNeu-Reln
InNeu-Sst
-log10 (q-value)
Log2 (FC)
L2-3
L2-4
L4
L5
L5-6-CC
L5-PT
L6
NRGN-1
NRGN-2
InNeu-Pv
InNeu-Sst
InNeu-Sv2c
InNeu-Vip
Apoe
Ubb
Gap43
Erbb4
Nrg1
Asic2
B
regulation of protein catabolic process
cytosolic ribosome
neurotransmitter transport
synapse organization
cation homeostasis
second-messenger-mediated signaling
regulation of membrane potential
cell part morphogenesis
presynapse
metal ion transmembrane transporter activity
cell-cell adhesion via plasma-membrane adhesion molecules
postsynapse
dendrite
synaptic signaling
axon
neuron migration
glutamate receptor activity
regulation of postsynaptic membrane potential
brain development
calcium ion binding
C
protein domain specific binding
developmental cell growth
main axon
cell adhesion molecule binding
protein localization to membrane
ATP metabolic process
primary active transmembrane transporter activity
regulation of ion transport
regulation of membrane potential
protein kinase binding
presynapse
brain development
cell junction organization
axon
synaptic signaling
dendrite
postsynapse
cell part morphogenesis
dendrite development
supramolecular fiber organization
D Human frontal cortex (hFCX)
Control
FTLD-GRN
PV
200µm
20µm
E Human frontal cortex (hFCX)
Cells / mm²
Synaptic density (puncta/µm²)
(n=5) (n=8)
F Human frontal cortex (hFCX)
DAPI PSD-95 LAMP1 MAP2
IMARIS
10µm
G Human frontal cortex (hFCX)
PSD-95⁺ LAMP1⁺
Surface Area in GFAP (µm²)
(n=4) (n=4)
H Human frontal cortex (hFCX)
DAPI PSD-95 IBA1 MAP2
I Human frontal cortex (hFCX)
NF68⁺
Intensity in GFAP
Intensity in Iba1
(n=4) (n=5)
J Human frontal cortex (hFCX)
NF68
100µm
K Human frontal cortex (hFCX)
White matter (WM)
300µm
L Human thalamus (hTH)
DAPI TDP-43 GFAP
M Human thalamus (hTH)
DAPI TDP-43 IBA1
N Human frontal cortex (hFCX)

Supplementary Figure 8. Characterizations of transcriptomic and phenotypic changes in the neuronal clusters in the thalamus and frontal cortex in *Grn*$^{-/-}$ mice and in FTLD-*GRN* patients. **A.** Volcano plots for the DEGs for neuronal clusters in mTH and mFCX from 19-month-old *Grn*$^{-/-}$ mice. **B-C.** Heatmap comparing the top 20 gene ontology (GO) terms defined by the DEGs in the neuronal clusters in the thalamus (B) and frontal cortex (C) of 19-month-old *Grn*$^{-/-}$ mice and FTLD-*GRN* patients. The color scale indicates -Log10 P-value calculated by the accumulative hypergeometric distribution. **D.** Immunohistochemical stains for parvalbumin (PV) in hFCX in control and FTLD-*GRN* patients. **E.** Quantification of PV$^+$ neurons and PV+ synaptic density in hFCX in controls and FTLD-*GRN* patients hFCX. Controls (n=5) and FTLD-*GRN* patients (n=8). Statistics uses Student's *t* test. **F.** Confocal and IMARIS images of PSD-95, LAMP1, and MAP2 showing colocalization of PSD-95$^+$ and LAMP1$^+$ vesicles in L2/3 of hFCX in control and FTLD-*GRN* patients. **G.** Quantification of PSD-95$^+$;LAMP1$^+$ area in GFAP$^+$ astrocytes in control and FTLD-*GRN* patients. Statistics use Student's *t* test. **H.** Confocal and IMARIS images of PSD-95$^+$ in IBA1$^+$ microglial processes and cell bodies in L2/3 of hFCX in control and FTLD-*GRN* patients. **I.** Quantification of NF68$^+$ intensity in GFAP$^+$ astrocytes (left) or IBA1$^+$ microglia (right) in control and FTLD-*GRN* patients. Statistics use Student's *t* test. **J-K.** Immunohistochemical stains for NF68 in hFCX L1/2 (J) and subcortical white matter (WM) (K) in control and FTLD-*GRN* patients. **L.** Confocal and IMARIS images of TDP-43 and GFAP in hTH in control and FTLD-*GRN* patients. **M-N.** Confocal and IMARIS images of TDP-43 and IBA1 in hTH (M) and L2/3 of hFCX (N) in control and FTLD-*GRN* patients. All quantification data represent mean $\pm$ SEM. * $P < 0.05$, ** $P < 0.01$.

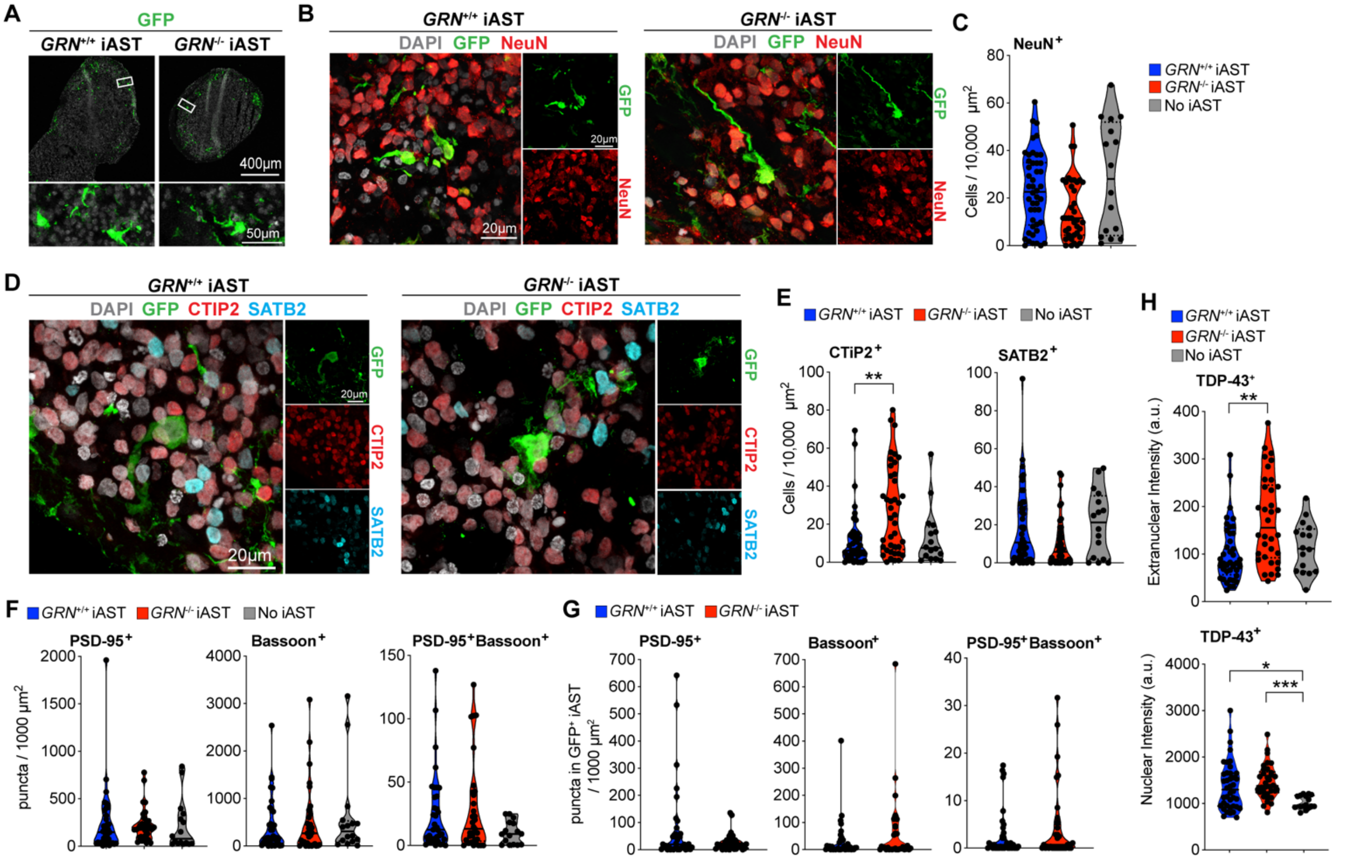

Supplementary Figure 9. Effects of *GRN*$^{+/+}$ and *GRN*$^{-/-}$ iAST on neuronal differentiation, synapse formation, and TDP-43 protein distribution in cortical organoids. A. Immunofluorescent images of GFP$^+$ *GRN*$^{+/+}$ iAST and GFP$^+$ *GRN*$^{-/-}$ iAST following transplantation in cortical organoids. **B.** Confocal images of GFP, NeuN, and DAPI in cortical organoids transplanted with *GRN*$^{+/+}$ iASTs or *GRN*$^{-/-}$ iASTs. **C.** Quantification of NeuN$^+$ cell density in cortical organoids transplanted with *GRN*$^{+/+}$ iASTs (n = 11), *GRN*$^{-/-}$ iASTs (n = 9), or no iASTs (n = 4). Statistics uses one-way ANOVA. **D.** Confocal images of GFP, CTiP2, and SATB2 in cortical organoids transplanted with *GRN*$^{+/+}$ iASTs or *GRN*$^{-/-}$ iASTs. **E.** Quantifications of CTiP2$^+$ and SATB2$^+$ cell density in cortical organoids transplanted with *GRN*$^{+/+}$ iASTs (n = 11), *GRN*$^{-/-}$ iASTs (n = 10), or no iASTs (n = 4). Statistics uses one-way ANOVA. **F.** Quantification of PSD-95$^+$, Bassoon$^+$, and PSD-95$^+$;Bassoon$^+$ puncta density in cortical organoids transplanted with *GRN*$^{+/+}$ iASTs (n = 10), *GRN*$^{-/-}$ iASTs (n = 9), or no iASTs (n = 4). Statistics uses one-way ANOVA. **G.** Quantification of PSD-95$^+$, Bassoon$^+$, and PSD-95$^+$;Bassoon$^+$ puncta density within the area of GFP$^+$ iAST in cortical organoids transplanted with *GRN*$^{+/+}$ iASTs (n = 10), *GRN*$^{-/-}$ iASTs (n = 9). Statistics uses Student's *t* test. **H.** Quantification of extranuclear (top) or nuclear (bottom) signal intensity of TDP-43 in organoids transplanted with *GRN*$^{+/+}$ iASTs (n = 11), *GRN*$^{-/-}$ iAST (n = 10), or no iASTs (n = 4). Statistics uses one-way ANOVA. All quantification data represent mean ± SEM. * $P < 0.05$, ** $P < 0.01$, *** $P < 0.001$.

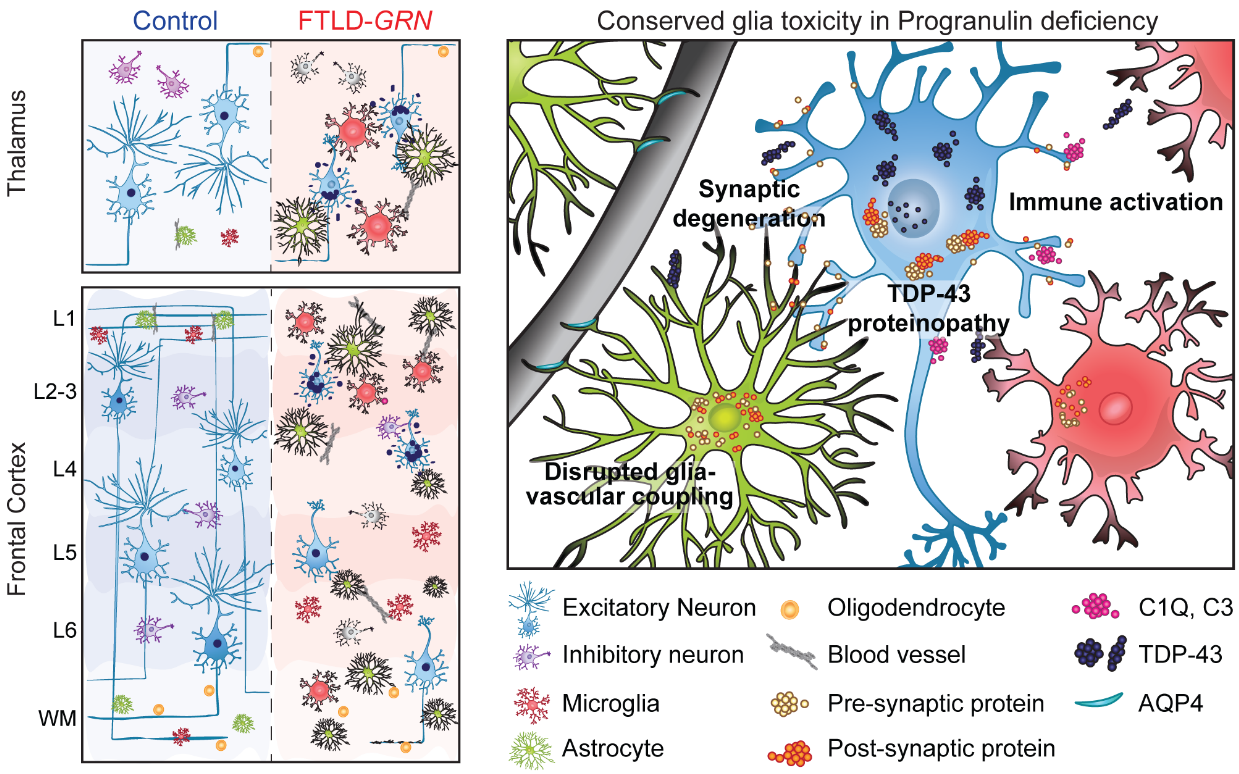

Supplementary Figure 10. Schematic diagrams showing the region-specific differences in microglial and astroglial pathology in the thalamus and frontal cortex in FTLD-*GRN* patients (left panels). Within the thalamus, both microglia and astrocytes exhibit transcriptomic and phenotypic changes similar to those observed in the thalamus of 19-month-old *Grn*$^{-/-}$ mice. These include microglia showing evidence of immune activation and astrocytes that disrupt glia-vascular coupling and promote synaptic degeneration (right panel).
