# Supplementary material for: Astroglial toxicity promotes synaptic degeneration in the thalamocortical circuit in frontotemporal dementia with GRN mutations
Source: J Clin Invest. 2023 Mar 15;133(6):e164919. doi: 10.1172/JCI164919 (PMC10014110; doi:10.1172/JCI164919)
Supplement: Supplemental tables 1 and 5 [file jci-133-164919-s062.pdf]

Supplementary Table 1 **Demographic information regarding control and FTLD-*GRN* patients (related to Figure 1 and 2)**

Sample ID	Origin	Sex	Age at onset (y)	Age at death (y)	Disease duration (y)	PMI (hrs)	Clinical	Mutation	MFG RIN	Thalamus RIN
Control 1	UCSF[a]	F	*NA*	68	NA	18	Control	*NA*	8.90	7.10
Control 2	UCSF[a]	M	*NA*	70	NA	36	Control	*NA*	8.70	9.30
Control 3	UCSF[a]	M	*NA*	54	NA	22	Control	*NA*	7.80	9.10
Control 4	UCSF[a]	M	*NA*	49	NA	28	Control	*NA*	7.90	9.30
Control 5	UCSF[a]	F	*NA*	88	NA	28	Control	*NA*	9.20	9.30
Control 6	UCSF[a]	F	*NA*	68	NA	11	Control	*NA*	8.70	6.60
Control 7	NIH[b]	F	*NA*	78	NA	24	Control	*NA*	7.50	8.30
Control 8	NIH[b]	F	*NA*	66	NA	10	Control	*NA*	8.10	9.30
Control 9	NIH[b]	M	*NA*	64	NA	8	Control	*NA*	7.10	7.70
Control 10	NIH[b]	F	*NA*	73	NA	3	Control	*NA*	9.10	8.80
Control 11	UCSF[a]	F	*NA*	72	NA	25	Control	*NA*	*NA*	7.10
FTLD-GRN 1	NDBB[c]	M	69	74	5	31	nfvPPA, CBS	c.1477C>T:p.Arg493*	8.00	6.10
FTLD-GRN 2	NDBB[c]	F	60	70	10	9	PPA, unspecified	c.709-2A>G	10.00	8.20
FTLD-GRN 3	NDBB[c]	F	47	59	12	10	CBS	c.1A>T:p.Met[1]	9.50	9.20
FTLD-GRN 4	NDBB[c]	F	51	56	5	8	bvFTD	c.1477C>T:p.Arg493*	9.90	7.70
FTLD-GRN 5	NDBB[c]	F	70	78	8	19	FTD, mixed	c.1216C>T:p.Gln406*	9.30	8.10
FTLD-GRN 6	NDBB[c]	F	65	73	8	7	bvFTD	c.328C>T:p.Arg110*	8.80	8.3
FTLD-GRN 7	NDBB[c]	M	59	64	5	7	bvFTD	c.264+2T>C	9.10	7.50
FTLD-GRN 8	NDBB[c]	F	60	66	6	7	bvFTD	c.347C>A:p.Ser116*	9.10	8.20
FTLD-GRN 9	NDBB[c]	F	63	73	10	21	nfvPPA, CBS	c.1145del:p.Thr382Serfs*30	8.50	7.30
FTLD-GRN 10	NDBB[c]	F	61	66	5	17	bvFTD	c.708+6_708+9del	*NA*	6.90

[a]UCSF Department of Pathology Autopsy Service
[b]NIH NeuroBioBank
[c]UCSF Neurodegenerative Disease Brain Bank

Supplementary Table 5. Information regarding antibodies used in this study.

ANTIBODIES	SOURCE	IDENTIFIER
Rabbit anti-ALDH1L1	Invitrogen (Cat# 702573, Recombinant monoclonal, 19H14L20, 1:500)	RRID: AB_2633093
Mouse anti-APOE	Abcam (Cat# ab1906, Monoclonal, D6E10, 1:100)	RRID: AB_302668
Rabbit anti-APOE	Sigma (Cat# HPA068768, Polyclonal, 1:150)	RRID: AB_2686029
Rabbit anti-Aquaporin 4	Sigma (Cat# HPA014784, Polyclonal, 1:5000)	RRID: AB_1844967
Rabbit anti-Bassoon	Synaptic Systems (Cat# 141-002, Polyclonal, 1:100)	RRID: AB_887698
Rabbit anti-BIN1	Abcam (Cat# ab182562, Monoclonal, EPR13463, 1:250)	RRID: N/A
Rabbit anti-C1Q	DAKO (Cat# F0254, Polyclonal, 1:500)	RRID: AB_2335713
Sheep anti-CD34	R&D Systems (Cat# AF7227, Polyclonal, 1:100)	RRID: AB_10973459
Rabbit anti-CLU	Abcam (Cat# ab92548, Monoclonal, EPR2911, 1:100)	RRID: AB_10585132
Rabbit anti-Connexin 43 (GJA1)	Cell Signaling (Cat# 3512, Polyclonal, 1:100)	RRID: AB_2294590
Rat anti-CTIP2	Abcam (Cat# ab18465, Monoclonal, 25B6, 1:500)	RRID: AB_2064130
Rabbit anti-EAAT1	Abcam (Cat# ab416, Polyclonal, 1:2000)	RRID: AB_304334
Rabbit anti-EAAT2	Abcam (Cat# ab41621, Polyclonal, 1:2000)	RRID: AB_941782
Goat anti-FTL	Abcam (Cat# ab110017, Polyclonal, 1:150)	RRID: AB_10861362
Rabbit anti-GAD1	Sigma (Cat# HPA058412, Polyclonal, 1:50)	RRID: AB_2683710
Rat anti-GFAP	Invitrogen (Cat# 13-0300, Monoclonal, 2.2B10, 1:100)	RRID: AB_2532994
Chicken anti-GFAP	Abcam (Cat# ab4674, Polyclonal, 1:2000)	RRID: AB_304558
Chicken anti-GFP	Aves Lab (Cat# 1020, Polyclonal, 1:500)	RRID: AB_10000240
Mouse anti-GLUR2/4	Chemicon (Cat# MAB396, Monoclonal, 3A11, 1:1000)	RRID: AB_94990

Rabbit anti-GPC5	Thermo Scientific (Cat# PA5-83490, Polyclonal, 1:200)	RRID: AB_2790643
Rabbit anti-HSPA1A	Enzo (Cat# ADI-SPA-812-D, Polyclonal, 1:100)	RRID: AB_11180512
Goat anti-IBA1	Novus Biologicals (Cat# NB100-1028, Polyclonal, 1:200)	RRID: AB_521594
Rabbit anti-KCNQ3	Alomore Labs (Cat# APC-051, Polyclonal, 1:200)	RRID: AB_2040103
Rabbit anti-LAMP1	Abcam (Cat#ab24170, Polyclonal, 1:300)	RRID: AB_775978
Chicken anti-MAP2	Abcam (Cat# ab5392, Polyclonal, 1:1000)	RRID: AB_2138153
Goat anti-MBP	Santa Cruz (Cat# SC-13914, Polyclonal, 1:2000)	RRID: AB_648798
Mouse anti-NeuN	Millipore (Cat# MAB377, Monoclonal, A60, 1:300)	RRID: AB_2298772
Mouse anti-Neurogranin	R&D Systems (Cat# MAB7947, Monoclonal, 898502, 1:10)	RRID: N/A
Mouse anti-NF68	Sigma (Cat# N5139, Monoclonal, NR4, 1:40)	RRID: AB_477276
Rabbit anti-Parvalbumin	Abcam (Cat# ab11427, Polyclonal, 1:400)	RRID: AB_298032
Rabbit anti-PGK1	Thermo Fisher (Cat# PA5-13863, Polyclonal, 1:50)	RRID: AB_2161216
Sheep anti-PGRN	R&D Systems (Cat# AF2557, Polyclonal, 1:50)	RRID: AB_2114504
Rabbit anti-PGRN	Sino Biological (Cat# 50396-RP02, Polyclonal, 1:1000)	RRID: N/A
Mouse anti-PSD95	Abcam (Cat# ab13552, Monoclonal, 7E3-1B8, 1:200)	RRID: AB_300453
Mouse anti-S100ß	Abcam (Cat# ab11178, Monoclonal, EP1576Y, 1:1000)	RRID: AB_297817
Mouse anti-SATB2	Abcam (Cat# ab51502, Monoclonal, SATBA4B10, 1:250)	RRID: AB_882455
Rabbit anti-SLC6A1	Sigma (Cat# HPA013341, Polyclonal, 1:200)	RRID: AB_1849509
Guinea Pig anti-Synaptophysin	Synaptic Systems (Cat# 101-004, Polyclonal, 1:200)	RRID: AB_1210382
Rabbit anti-TDP-43 (N-term)	Protein Tech (Cat# 10782-2AP, Polyclonal, 1:300)	RRID: AB_615042
Secondary antibodies		

Donkey anti-Chicken 488	Jackson (Cat# 703-545-155, Polyclonal)	RRID: AB_2340375
Donkey anti-Chicken 633	Biotium (Cat# 20168, Polyclonal)	RRID: AB_10853143
Donkey anti-Goat 488	Invitrogen (Cat# A11055, Polyclonal)	RRID: AB_2534102
Donkey anti-Goat 568	Invitrogen (Cat# A11057, Polyclonal)	RRID: AB_142581
Donkey anti-Mouse 488	Invitrogen (Cat# A21202, Polyclonal)	RRID: AB_141607
Donkey anti-Mouse 568 (H+L)	Invitrogen (Cat# A10037, Polyclonal)	RRID: AB_2534013
Donkey anti-Mouse 647	Invitrogen (Cat# A31571, Polyclonal)	RRID: AB_162542
Donkey anti-Rabbit 488	Invitrogen (Cat# A21206, Polyclonal)	RRID: AB_2535792
Donkey anti-Rabbit 568	Invitrogen (Cat# A10042, Polyclonal)	RRID: AB_2534017
Donkey anti-Rabbit 647	Invitrogen (Cat# A31573, Polyclonal)	RRID: AB_2536183
Donkey anti-Rat 594	Invitrogen (Cat# A21209, Polyclonal)	RRID: AB_2535795
Donkey anti-Rat 647	Abcam (Cat# ab150155, Polyclonal)	RRID: AB_2813835
Donkey anti-Sheep 488	Invitrogen (Cat# A11015, Polyclonal)	RRID: AB_141362
Donkey anti-Sheep 568	Invitrogen (Cat# A21099, Polyclonal)	RRID: AB_2535753
Goat anti-Guinea Pig 568	Invitrogen (Cat# A11075, Polyclonal)	RRID: AB_141954
Goat anti-Mouse 568	Invitrogen (Cat# A11004, Polyclonal)	RRID: AB_2534072
Goat anti-Rabbit 488	Invitrogen (Cat# A11008, Polyclonal)	RRID: AB_143165
Goat anti-Chicken (H+L) biotinylated	Vector Laboratories (Cat# BA-9010)	RRID: AB_2336114
Goat anti-Mouse (H+L) biotinylated	Vector Laboratories (Cat# BA-9200)	RRID: AB_2336171
Goat anti-Rabbit (H+L) biotinylated	Vector Laboratories (Cat# BA-1000)	RRID: AB_2313606
Rabbit anti-Goat (H+L) biotinylated	Vector Laboratories (Cat# BA-5000)	RRID: AB_2336126
Rabbit anti-Sheep (H+L) biotinylated	Vector Laboratories (Cat# BA-6000)	RRID: AB_2336217
